# Spin Crossover in a Hexaamineiron(II) Complex: Experimental Confirmation of a Computational Prediction

**DOI:** 10.1002/chem.201705439

**Published:** 2018-01-29

**Authors:** Paul V. Bernhardt, Jessica K. Bilyj, Victor Brosius, Dmitry Chernyshov, Robert J. Deeth, Marco Foscato, Vidar R. Jensen, Nicole Mertes, Mark J. Riley, Karl W. Törnroos

**Affiliations:** ^1^ School of Chemistry and Molecular Biosciences University of Queensland Brisbane 4072 Australia; ^2^ European Synchrotron Radiation Facility 71 Avenue des Martyrs Grenoble 38000 France; ^3^ Department of Chemistry University of Warwick Coventry CV4 7AL UK; ^4^ Department of Chemistry University of Bergen Allégaten 41 5007 Bergen Norway; ^5^ Present address: Department of Chemistry University of Bath, Claverton Down Bath BA2 7AY UK

**Keywords:** amines, density functional calculations, iron, optical spectroscopy, spin crossover

## Abstract

Single crystal structural analysis of [Fe^II^(tame)_2_]Cl_2_⋅MeOH (tame=1,1,1‐tris(aminomethyl)ethane) as a function of temperature reveals a smooth crossover between a high temperature high‐spin octahedral *d* ^6^ state and a low temperature low‐spin ground state without change of the symmetry of the crystal structure. The temperature at which the high and low spin states are present in equal proportions is *T*_1/2_=140 K. Single crystal, variable‐temperature optical spectroscopy of [Fe^II^(tame)_2_]Cl_2_⋅MeOH is consistent with this change in electronic ground state. These experimental results confirm the spin activity predicted for [Fe^II^(tame)_2_]^2+^ during its de novo artificial evolution design as a spin‐crossover complex [*Chem. Inf. Model*. **2015**, *55*, 1844], offering the first experimental validation of a functional transition‐metal complex predicted by such in silico molecular design methods. Additional quantum chemical calculations offer, together with the crystal structure analysis, insight into the role of spin‐passive structural components. A thermodynamic analysis based on an Ising‐like mean field model (Slichter–Drickammer approximation) provides estimates of the enthalpy, entropy and cooperativity of the crossover between the high and low spin states.

Spin crossover (SCO) in molecular materials is associated with a change of spin state of a central 3*d* metal under external perturbation such as temperature, pressure and irradiation.^1^ The spin state instability is expected for predominantly octahedral complexes of 3*d* metals where the LS ground state and the metastable HS excited state are close in energy but differ in terms of entropy contributions in their free energies.^2^ All macroscopic properties conjugated with the spin state, such as a colour, magnetization, density, and sometimes even shape and size of the crystals may be altered by external perturbation. Therefore, SCO compounds may be candidate materials for applications including sensors, imaging and information storage.^3^

The potential applications make rational and computational design of SCO materials attractive, albeit very challenging, goals due to the delicate energetics of the spin states involved.^4^ Spin instability in an isolated complex is well understood already within ligand field theory^5^ and the collective behaviour of spin‐active molecules can be parameterized with Ising‐like models^6^ and a generic phase diagram is provided by Landau theory.^7^

Although quantum chemical methods such as density functional theory (DFT) may predict the relative stability of the spin states with useful accuracy, these methods are computationally too expensive for materials design. In contrast, even simple correlations based on geometry parameters may sometimes accurately predict the relative spin state stability of a spin‐active complex,^8^ albeit only within relatively narrow ligand classes. A broader applicability domain is offered by ligand‐field molecular mechanics (LFMM).^9^ LFMM is molecular mechanics augmented by empirical terms to treat the *d*‐orbital splitting and interelectronic repulsion, and may predict relative spin‐state stability with an accuracy approaching that of DFT, only orders of magnitude faster.^10^ This accuracy has been used to demonstrate that LFMM can be applied in structural screening to identify SCO candidate complexes4b and as the fitness function (figure of merit, or scoring function) in de novo design of candidate Fe^II^ SCO complexes.^11^ Although the most promising candidates were predicted by DFT to be bistable, computationally designed SCO compounds have yet to be tested experimentally.

Experimental validation will help determine to what extent molecular computational design including the local spin‐active complex alone may be useful in development of SCO materials. In this report we present the case of [Fe^II^(tame)_2_]Cl_2_⋅MeOH [Scheme 1] (tame=1,1,1‐tris(aminomethyl)ethane). The complex cation [Fe^II^(tame)_2_]^2+^ emerged as the most promising candidate predicted by the above‐mentioned de novo design study.^11^

**Scheme 1:**
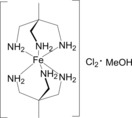
Structure of [Fe^II^(tame)_2_]Cl_2_ (tame=1,1,1‐tris(aminomethyl)ethane) as its MeOH solvate.

The properties of solid SCO materials are far more complex than those of isolated spin‐active fragments. For example, spin‐passive structural components not directly bonded to the transition‐metal complex, such as solvent molecules or counter ions, may affect the thermodynamics of the transition.^12^ This level of complexity is currently beyond routine theoretical predictions. Screening4b and de novo design^11^ based on the central complex alone thus serve as a guide toward the Fe^II^ complexes most likely to lead to SCO behaviour subject to outer‐sphere perturbations. Therefore, experimental studies of crystal structures and the empirical correlations between various packing modes and collective responses are essential to understand the mechanism of propagation of a spin‐switch throughout the crystal. In this work spin crossover is reflected not only in the Fe−N bond distances but also in the size and shape of the unit cell, unit cell volume and optical properties. These data are used to estimate basic thermodynamics parameters, such as enthalpy, entropy, and cooperativity, based on the mean‐field free energy expression.^13^

There are relatively few ligand systems that have been shown to form hexaamine complexes with Fe^II^ (Fe^II^N_6_). A selection of these are shown in Scheme 2. Iron(II) complexes of organic amines are typically air sensitive and once oxidized to their ferric form usually undergo Fe‐catalysed oxidative ligand dehydrogenation to generate unsaturated imine ligands.^14^ This ligand degradation reaction can be avoided if the geometric constraints of the ligand prevent dehydrogenation of the Fe‐N(H)‐C(H) bond as in the macrocyclic complexes [Fe^II^(tacn)_2_]^2+^ and [Fe^II^(*trans*‐diammac)]^2+^ (Scheme 2) where stable ferrous^15^ and ferric^16^ complexes have been isolated and structurally characterized.

**Scheme 2:**
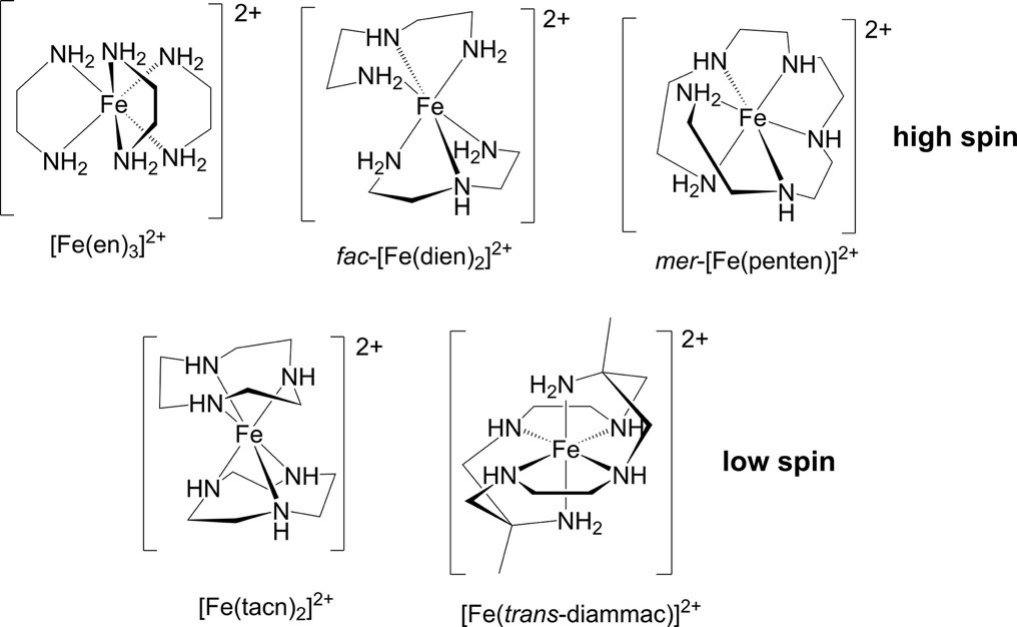
A selection of crystallographically characterised hexaamineiron(II) complexes.

This presented a challenge in isolating the [Fe^II^(tame)_2_]^2+^ complex in a pure form. Both oxygen and water were problematic. If oxygen is present, immediate oxidation of the mixture to give an insoluble precipitate of ferric oxide results, probably coupled with ligand dehydrogenation. Secondly, unless anhydrous MeOH is employed, addition of the tame free base leads to formation of metal hydroxides. Thirdly, the desired product [Fe^II^(tame)_2_]Cl_2_⋅MeOH must be crystallised by slow addition of chloride to a solution of ferrous triflate and tame free ligand followed by slow evaporation of the MeOH solution. If chloride is present in a stoichiometric amount during Fe^II^/tame complexation then the reaction does not yield a crystalline product. For these reasons, high yields of pure compound were not pursued and the crystalline product was separated from other material manually under a microscope. In spite of its instability in solution and susceptibility to oxidation, in the solid state the crystals are robust (for more than a year) if protected from oxygen and free from solvent. Furthermore, crystals of [Fe^II^(tame)_2_]Cl_2_⋅MeOH can be manipulated in air for periods of hours without any noticeable degradation.

The crystal structure of [Fe^II^(tame)_2_]Cl_2_⋅MeOH was determined at a number of temperatures from 10 K to 333 K. In all cases a trigonal structure was defined (space group *R* $\bar{3}$ m), which is isomorphous with [Ni^II^(tame)_2_]Cl_2_.^17^ The complex cation occupies a site with $\bar{3}$ m (*D*_3*d*_) symmetry. The methyl and quaternary C‐atoms lie on a threefold axis (coinciding with three vertical mirror planes). An initial refinement found the methylene and amine groups of the ligand on a crystallographic mirror plane, as reported for the Ni^II^ analogue.^17^ However, abnormal thermal parameters and an unreasonably short H_2_N‐CH_2_ bond length (1.433(5) Å) indicated that the N‐atom was actually displaced from the mirror plane and disordered either side as are the methylene protons. Indeed, DFT calculations (see Supporting Information) confirm that the [Fe^II^(tame)_2_]^2+^ cation gains stability by twisting each tripodal “cap” about its *C*_3_ axis in either direction, thus removing the mirror plane symmetry elements. The disorder is fixed by symmetry and there seems to be no ordering process correlated with spin crossover at least at the level of the average structure. So the structure amounts to a disordered mixture of *S*_6_‐symmetric complex cations related by a *C*_2_ symmetry operation (see Supporting Information, Figure S1). The crystal structure is built from layers of cationic spin‐active Fe complexes and Cl^−^ counter‐ions, the layers are separated by layers of disordered MeOH molecules; all the layers are orthogonal to the (threefold) ***c***‐axis (Figure 1, right).

**Figure 1:**
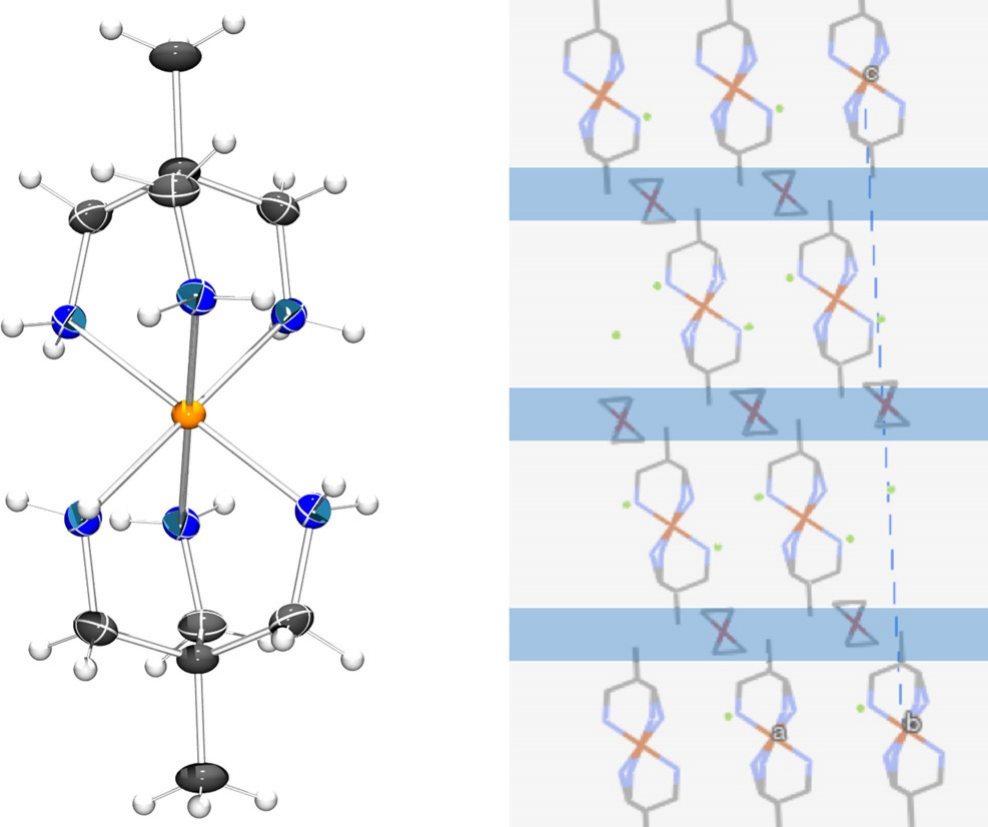
Left panel: ORTEP view (rendered with PovRay) of the [Fe^II^(tame)_2_]^2+^ cation (300 K) showing its true molecular *S*_6_ symmetry. The right panel shows the packing scheme: electroneutral layers of [Fe^II^(tame)_2_]Cl_2_ in the **a b** plane are separated by layers of disordered MeOH molecules.

Spin inversion (LS→HS) is associated with elongation of Fe−N bonds by ≈0.15 Å. This deformation mainly affects the *c*‐axis of the unit cell, which is elongated by more than 3.5 %, while the ***a***‐axis is less affected (≈0.5 % elongation). This reflects an anisotropy of the packing forces with stronger Coulomb interactions within the anion–cation layers and weaker between the layers.

The elongation of ***c***, increase in unit cell volume, and increase in the apparent Fe−N bond length are all correlated. The two extreme Fe−N bond lengths at 333 K (2.189(3) Å) and 10 K (2.035(3) Å) are consistent with high spin^18^ and low spin^15^ hexaamineiron(II) complexes, respectively. In the temperature range 250–333 K there is very little variation in the Fe−N coordinate bond lengths or angles. Similarly, the structures in the low temperature range 10–60 K are essentially the same and consistent with a 100 % low spin Fe^II^ structure. In the intermediate range (60–250 K) a sigmoidal variation in the apparent Fe−N bond length as a function of temperature is seen, which reflects a weighted average of the relative proportions of HS and LS Fe^II^ in the crystal (Supporting Information Eqn S2).

The LS→HS spin‐transition‐induced elongation along the *C_3_* axis is qualitatively consistent with that predicted by previous molecular‐level modeling of [Fe^II^(tame)_2_]^2+^.^11^ DFT was used to quantify the elongation in models of the [Fe^II^(tame)_2_]^2+^ cation with and without the chloride counter ions (see Supporting Information). The results suggest that the [Fe^II^(tame)_2_]^2+^ complex engages in a network of NH⋅⋅⋅Cl interactions that are stronger in the more compact LS coordination. These interactions thus also limit the LS→HS transition‐induced elongation compared to that of Fe^II^N_6_ complexes of other ligands than tame.

The polarised single crystal visible spectrum at 11 K is shown in Figure 2 using light propagating perpendicular to the ***c*** axis with the polarisation ∥ ***c*** (π spectrum) and ⊥ ***c*** (σ spectrum) and unpolarised propagating parallel to the ***c*** axis (α spectrum). The relationship *σ*=α≠π implies that the transitions are electric dipole allowed in this direction and consistent with threefold‐symmetry of the complex cation (see Supporting Information for more details).

**Figure 2:**
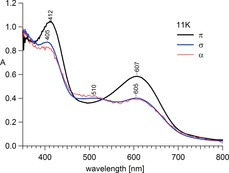
Polarised single crystal absorption spectra of [Fe^II^(tame)_2_Cl_2_]⋅MeOH.

The temperature dependent behaviour of the spectra (π‐polarisation, Figure 3 a), illustrates the spin‐crossover evolution between 50 K and 295 K as the intensity of the LS ^1^A_1g_→^1^T_1g_, ^1^T_2g_ transitions (O_h_ term symbols) decrease with increasing temperature. Figure 3 b shows the intensity of the 607 nm peak of the ^1^A_1g_→^1^T_1g_ transition in π‐polarisation for several cooling and heating cycles.

**Figure 3:**
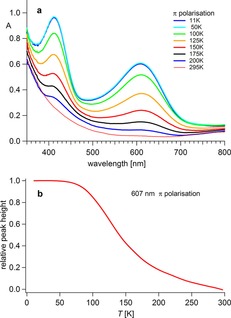
(a) Temperature dependent single crystal absorption spectra of [Fe^II^(tame)_2_Cl_2_]⋅MeOH (π‐polarisation) and (b) plot of the absorption at 607 nm as a function of temperature.

Entropy, enthalpy and cooperativity have been estimated on the basis of diffraction data using the Slichter–Drickamer approximation^13^ (see Figure 4 and the Supporting Information for details). Our estimates reveal that the cooperativity parameter for correlations between neighbouring spin centres is negative, indicating alternation of different states to be preferred over clustering of similar species.

**Figure 4:**
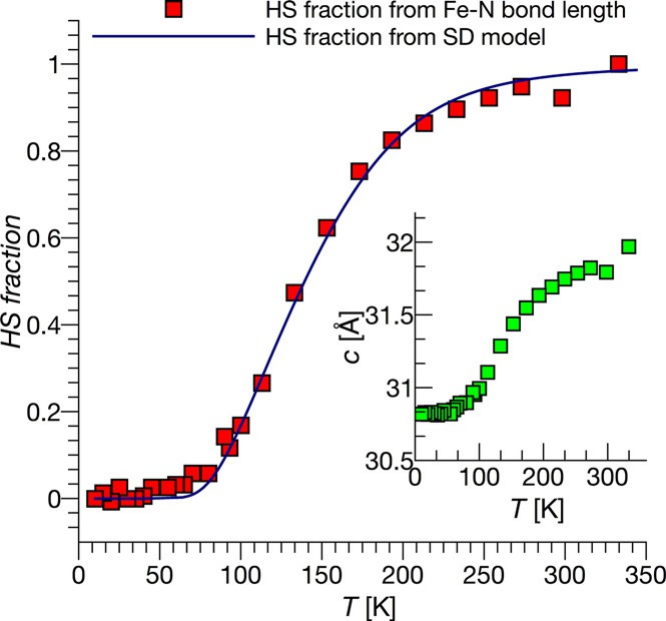
HS state fraction as a function of temperature. The squares indicate the diffraction data, the line corresponds to the Slichter‐Drickamer (SD) model with *Γ*=−500 J mol^−1^, Δ*H*=2300 J mol^−1^, Δ*S*=16.5 J mol^−1^ (see eqn S1): inset–the temperature dependence of the ***c***‐axis (Å).

Single crystal structural analysis and variable‐temperature optical spectroscopy have confirmed the spin‐crossover properties of [Fe^II^(tame)_2_]Cl_2_⋅MeOH. Since no superstructure has been observed, the title compound may serve as a candidate for observation of short‐range correlations, associated diffuse scattering, and other non‐linear phenomena near *T*_1/2_. Importantly, the central cation [Fe^II^(tame)_2_]^2+^ was automatically designed from scratch by assembling small and unconstrained structural fragments (in silico de novo molecular design) to give Fe^II^N_6_ complexes. These complexes were optimized (by artificial evolution) to express spin bistability.^11^ To our knowledge, such an automated de novo procedure is here, for the first time, experimentally confirmed to have predicted a compound that reflects the intended, in silico optimised property. This is a promising step forward for de novo molecular design beyond traditional drug‐like organic molecules.

## Experimental Section

Syntheses, instrumental and computation details are in the Supporting Information.

## Conflict of interest

The authors declare no conflict of interest.

## Supporting information

As a service to our authors and readers, this journal provides supporting information supplied by the authors. Such materials are peer reviewed and may be re‐organized for online delivery, but are not copy‐edited or typeset. Technical support issues arising from supporting information (other than missing files) should be addressed to the authors.

Supplementary
