# Supplementary material for: Spin Crossover in a Hexaamineiron(II) Complex: Experimental Confirmation of a Computational Prediction
Source: Chemistry. 2018 Jan 29;24(20):5082–5. doi: 10.1002/chem.201705439 (PMC5988046; doi:10.1002/chem.201705439)
Supplement: Supplementary file 1 — Supplementary [file CHEM-24-5082-s001.pdf]

CHEMISTRY
A European Journal

Supporting Information

Spin Crossover in a Hexaamineiron(II) Complex: Experimental Confirmation of a Computational Prediction

Paul V. Bernhardt,*[a] Jessica K. Bilyj,[a] Victor Brosius,[a] Dmitry Chernyshov,*[b] Robert J. Deeth,[c, e] Marco Foscato,[d] Vidar R. Jensen,*[d] Nicole Mertes,[a] Mark J. Riley,*[a] and Karl W. Törnroos*[d]

chem_201705439_sm_miscellaneous_information.pdf

Table of Contents

Experimental **S2**
Synthesis **S2**
tame (free base) S2
$[Fe(tame)_2]Cl_2 \cdot MeOH$ S2
Optical Spectroscopy **S2**
X-ray Crystallography **S2**

Results **S4**
X-Ray crystallography **S4**
Optical spectroscopy **S5**
Theoretical Spin-Transition Model **S6**
Molecular-Level Computational Chemistry **S7**
Computational Details S7
Conformations of $[Fe(tame)_2]^{2+}$ S8
The Structure and LS-HS Energy Gap of the Iron Complex S9
Cartesian coordinates of DFT-optimized geometries S12

References **S25**

Experimental

Synthesis

Tris(aminomethane)ethane trihydrochloride (tame.3HCl) was prepared as described.[1] MeOH was dried over 3Å molecular sieves and stored in an anaerobic box. All other reagents were obtained commercially.

tame (free base)

Tris(aminomethane)ethane trihydrochloride (5.42 g, 24.14 mmol, 1 eq) was suspended in toluene (30 mL) and KOH (10.83 g, 193.09 mmol, 8 eq) was added. The mixture was stirred for 2 h under reflux and the toluene decanted to another flask. This procedure was done two more times (but no further KOH was added). The three toluene fractions were combined and the solvent was removed under reduced pressure. Distillation under reduced pressure (high vacuum) afforded 1.50 g (12.80 mmol, 53 %) of a colourless oil of tris(aminomethyl)ethane. ^{1}H-NMR (D_2O, δ [ppm]): 0.83 (s, 3 H, CH_3), 2.49 (s, 6 H, CH_2).

[Fe(tame)$_2$]Cl$_2$·MeOH

In a glovebox under a nitrogen atmosphere (O_2 < 20 ppm) tame free ligand (50 mg, 426.65 µmol, 2 eq) and ferrous triflate ($Fe(OTf)_2$) (75.51 mg, 213.32 µmol, 1 eq) were mixed in dry and oxygen free MeOH (10 mL). The solution was gravity filtered if necessary to remove any unwanted precipitate. A solution of tetraethylammonium chloride (70.70 mg, 426.65 µmol, 2 eq) in 10 mL MeOH was added to the filtrate gradually, via a syringe pump, over 24 h. Upon concentration of the solution, colourless trigonal crystals of the desired product formed over the course of a few days. Upon complete evaporation of the solvent these crystals were separated from any impurities by hand picking (in air) under a microscope.

Optical Spectroscopy

Single crystal absorption spectra were obtained using an oriented crystal ~200×200 µm mounted on a copper mask inside a close-cycle cryostat (Leybold). Light from a Tungsten lamp was dispersed through a 0.75m monochromator (Jobin Yvon 750S), chopped (New Focus 3501), polarised (calcite) and passed through the sample and focussed on an avalanche photodiode (Advanced Photonix, 5mm). The signal was acquired using lock-in detection (Stanford SRS830). Instrument control and acquisition were using GPIB protocols and Labview software.[2]

X-ray Crystallography

Three series of single crystal diffraction experiments were carried out. First, the data were collected with an Oxford Diffraction Gemini CCD diffractometer employing graphite-monochromated Mo-Kα radiation (0.71073 Å) and operating within the range $2 < 2\theta < 50$ Å. Crystals were cooled or heated with an Oxford Cryosystems 600 series Cryostream Cooler or Desktop Cooler. Data reduction and empirical absorption corrections (multi-scan) were performed with Oxford Diffraction CrysAlisPro software (Oxford Diffraction, vers. 171.36.21). More complete data have been collected with PILATUS@SNBL diffractometer at the Swiss

Norwegian Beam Line, BM01 end station of the European Synchrotron Radiation Facility, Grenoble, France[3]. The temperature was controlled with CryoStream 700 Plus cooler (Oxford Cryosystems) in the range 90-300 K, using an energy (from double Si(111) focussing mirror) of 17.715 EV (0.69999 Å) in 16 bunch mode. A third series of measurements has been also done with the PILATUS@SNBL diffractometer with a CryoCool He cooler (Cryoindustries of America) in the range 10-100 K using an energy of 18.357 EV (0.67540 Å) in 7/8 multi bunch mode. The synchrotron data were pre-processed with SNBL ToolBox[3] and processed with CrysAlis software (Rigaku Oxford Diffraction). The structure was solved with SHELXT and refined with SHELXL.[4] The experimental data and results of the structure analysis are given in Table 1. The molecular structure diagram was produced with ORTEP3.[5] Crystallographic data in CIF format have been deposited with CCDC numbers 1585457-1585483.

Results

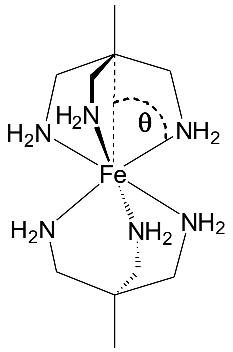

Chart S1: Definition of θ (acute angle between the C_3 axis and The Fe-N vector). For ideal octahedral symmetry θ = 54.74°.

X-Ray crystallography

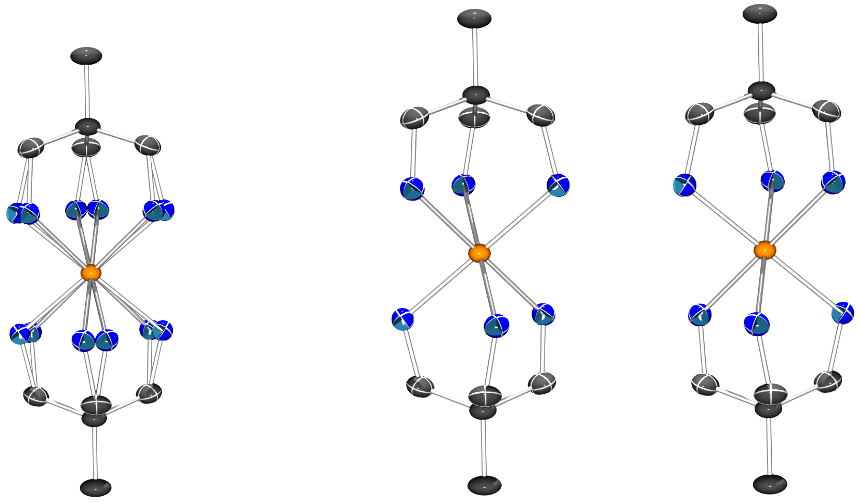

Figure S1: Disorder model in the $[Fe(tame)_2]^{2+}$ cation (H-atoms omitted). (left) the D_{3d} symmetric disordered cation with the vertical mirror plane perpendicular to the plane of the page (right) The two components of the disorder model (S_6 symmetry) which are identical but related by a C_2 axis perpendicular to the principal C_3 axis.

Table S1: Temperature dependent structural parameters for $[Fe(tame)_2]Cl_2{\cdot}MeOH$

T (K)	*a* (Å)	*c* (Å)	*V* (Å³)	Fe-N (Å)	θ (°)
10	7.1738(5)	30.815(2)	1373.4(2)	2.035(3)	53.4
15	7.1744(4)	30.829(2)	1374.3(2)	2.037(3)	53.4
20	7.1739(4)	30.816(2)	1373.5(2)	2.034(3)	53.4
25	7.1726(6)	30.830(2)	1373.6(3)	2.039(4)	53.3
30	7.1740(6)	30.822(2)	1373.8(3)	2.034(4)	53.3
35	7.1745(5)	30.813(2)	1373.6(2)	2.035(3)	53.4
40	7.1745(5)	30.820(2)	1373.9(3)	2.036(3)	53.4
45	7.1745(5)	30.843(2)	1374.9(2)	2.039(3)	53.3
55	7.1745(5)	30.820(2)	1373.9(2)	2.039(3)	53.5
60	7.1743(5)	30.848(2)	1375.0(2)	2.040(3)	53.5
65	7.1753(5)	30.867(2)	1376.3(2)	2.040(3)	53.4
70	7.1740(5)	30.893(2)	1376.9(2)	2.044(3)	53.4
80	7.1755(5)	30.896(2)	1377.7(2)	2.044(3)	53.4
90	7.1733(5)	30.966(2)	1379.9(2)	2.057(3)	53.1
93	7.1815(8)	30.952(4)	1382.5(4)	2.053(3)	53.2
100	7.1732(5)	30.993(2)	1381.1(2)	2.061(3)	53.1
113	7.176(1)	31.104(5)	1386.9(4)	2.076(3)	52.9
133	7.1724(8)	31.284(3)	1393.7(4)	2.108(3)	52.6
153	7.1728(8)	31.436(3)	1400.7(3)	2.131(3)	52.3
173	7.1748(8)	31.545(3)	1406.3(4)	2.151(3)	52.1
193	7.1786(6)	31.631(3)	1411.6(2)	2.162(3)	51.9
213	7.1799(5)	31.689(2)	1414.8(2)	2.168(3)	51.8
233	7.1845(5)	31.743(2)	1418.9(2)	2.173(3)	51.7
253	7.1900(4)	31.784(2)	1423.0(2)	2.177(3)	51.7
273	7.1953(4)	31.819(2)	1426.7(2)	2.181(3)	51.7
298	7.1854(5)	31.790(2)	1421.4(2)	2.177(2)	51.6
333	7.2204(5)	31.965(3)	1443.2(2)	2.189(3)	51.5

Optical spectroscopy

The symmetry of the centrosymmetric $Fe^{II}N_6$ chromophore is not octahedral but is best represented by the D_{3d} point group. Figure S2 shows a Tanabe Sugano diagram for HS and LS $[Fe(tame)_2]^{2+}$ in this point group. The coordinate that best illustrates the departure from octahedral symmetry is the angle θ defined in Chart S1; the acute angle between the Fe–N vector and the C_3 axis. For an ideal octahedral complex θ = 54.74° while θ = 53.4° for the LS form (Fe–N = 2.035 Å) and θ = 51.5° for the HS structure (Fe–N = 2.189 Å). In other words the complex stretches along its C_3 axis during the LS→HS transition and the N–Fe–N angles within each cap contract but the point symmetry remains the same.

The octahedral part of the ligand field is quantified using angular overlap parameters assuming $e_\pi = e_\sigma /4$; a value of e_σ=9650 cm^{-1} at the LS geometry and a bond length dependence given by $e_\sigma(R) \propto r^{-6}$. The Racah parameters B=638 cm^{-1};

C=3742 cm^{-1} were taken from the work of Hauser[6] who observed the LS $^1A_{1g} \rightarrow$ $^3T_{1g}$, $^3T_{2g}$ spin-forbidden transitions in $[Fe(ptz)_6](BF_4)_2$ (ptz = 1-propyltetrazole). The trigonal elongation in $[Fe(tame)_2]Cl_2{\cdot}MeOH$ results in a splitting of the $^1T_{1g}$ and $^1T_{2g}$ states (Figure S2). The splitting of the higher energy $^1T_{2g}$ (O_h) state is larger than the $^1T_{1g}$ state. The higher energy component of the split $^1T_{2g}$ state is calculated to be ^1E_g, and the intensity changes as a function of the direction of polarisation (Figure 3) are in line with the selection rules that dictate that the $^1A_{1g}{\rightarrow}^1E_g$ (D_{3d}) transitions are electric dipole allowed in σ polarisation. The introduction spin-orbit coupling to the calculations had no significant effect. There is an additional small peak at 510 nm in σ and α polarisation seen at low temperature (Figure 3). The origin of this transition is unclear, but must be due to one or more spin-forbidden transitions that are calculated to be in this region (Figure S2).

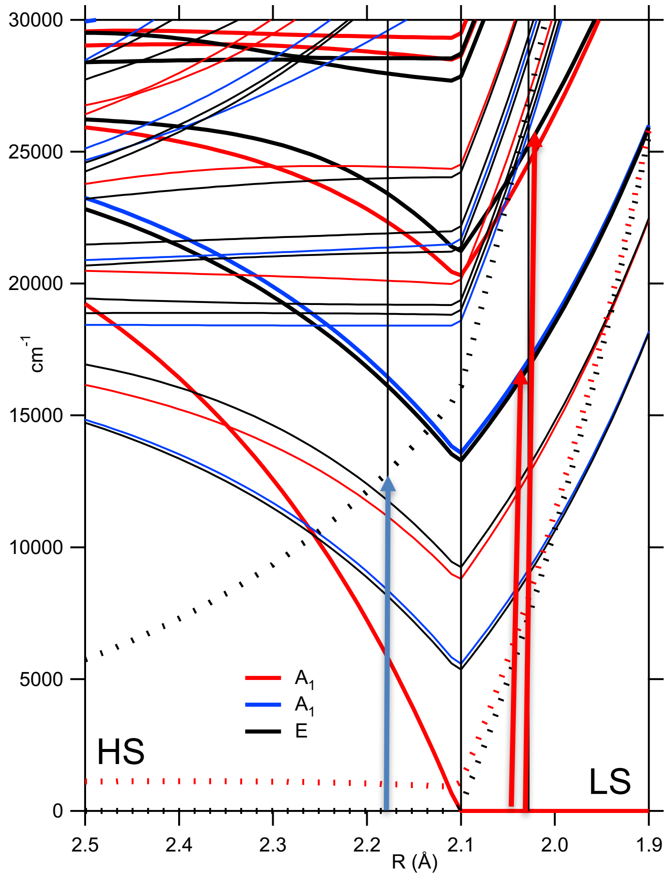

Figure S2: Ligand field calculation in D_{3d} symmetry assuming, $e_\pi = e_\sigma/4$, e_σ=9650 cm^{-1} at the LS geometry and $e_\sigma(R) \propto 1/r^6$. Racah parameters B=638 cm^{-1}; C=3742 cm^{-1}, taken from the literature[6] (zero spin-orbit coupling); heavy, light and dotted lines correspond to singlet, triplet and quintet states. The spin allowed transitions are indicated by the arrows.

Theoretical Spin-Transition Model

Spin transition scenarios are frequently modelled with the so-called Slichter-Drickamer approximation for the free energy of the spin crossover system.[7] This

approach stems from an Ising-like lattice model adapted for spin-crossover,[8] the basic formulae of which are given below. A crystallographic site occupied by a spin-active complex can be found in either a LS or HS ground state. Considering a mixture of such states in a crystal as a regular solid solution, one arrives at the following expression for the Gibbs energy:

$$F = \gamma(\Delta H - RT\Delta S) + \Gamma\gamma(1-\gamma) - RT(\gamma \ln\gamma + (1-\gamma)\ln(1-\gamma)) \qquad (S1)$$

Here Γ is a measure of cooperativity (a phenomenological constant), and γ stands for the fraction of HS complexes, which is obtained from the experimental crystallographically determined apparent (averaged) Fe-N bond lengths:

$$\gamma(\mathrm{T}) = \frac{\mathrm{d_{Fe-N}(T) - d_{Fe-N}(10K)}}{\mathrm{d_{Fe-N}(300K) - d_{Fe-N}(10K)}} \qquad (S2)$$

It is thereby assumed that at room temperature 100% of the complexes are HS and at low temperature (10 K) all complexes are in the LS state. The terms ΔH and ΔS in eqn (S1) denote entropy and enthalpy costs associated with the spin-state change. The second term accounts for the cooperative interactions and the last one represents the mixing entropy. Minimization of the above expression gives the link between the HS fraction at equilibrium and temperature:

$$\mathrm{T} = \frac{\Delta \mathrm{H} + \Gamma(1\text{-}2\gamma)}{\Delta \mathrm{S} - \mathrm{R}\ln(\frac{\gamma}{1-\gamma})} \qquad (S3)$$

This expression has been used to estimate thermodynamic parameters from the spin fraction. At the temperature ($T_{½}$) where the HS and LS forms are equally abundant (γ= ½), eqn (S3) simplifies to eqn (S4).

$$\mathrm{T}_{½} = \frac{\Delta \mathrm{H}}{\Delta \mathrm{S}} \qquad (S4)$$

Molecular-Level Computational Chemistry

Computational Details

All density functional theory (DFT) calculations were performed with the Gaussian 09 program, version D.01,[9] using the thus-implemented B3LYP,[10] OPBE,[11] PBE,[11b, 12] and BP86[13] functionals. The B3LYP, PBE and BP86 functionals were corrected by the D3-version of Grimme's D3 empirical dispersion.[14] The dispersion term included Becke–Johnson (BJ) damping with revised parameters derived from a thorough re-parametrization involving large training sets including short interatomic distances. [15] The resulting dispersion term is here termed D3MBJ and is added to the B3LYP, PBE and BP86 functionals to give the corresponding dispersion-corrected functionals DFT-D3MBJ.

The OPBE functional was used here specifically to address the energy gap between high-spin and low-spin states. In doing so, we used an overall computational model and protocol similar to that of previous work in which this functional has been used to reproduce the stability of spin states with good accuracy.[16] Thus, the OPBE

functional was used in conjunction with implicit solvation in water using the polarizable continuum model (PCM) as implemented in Gaussian 09. Non-electrostatic contributions, i.e., solute-solvent dispersion, repulsion interaction energies, and the solute cavitation energy, were included.[17]

All DFT calculations were performed using Dunning's correlation-consistent and polarized valence basis sets; either triple-ζ (cc-pVTZ; for models with or without counterion using OPBE, PBE , or BP86) or quadruple-ζ (cc-pVQZ; when comparing conformations using B3LYP).[18] Numerical integration was performed using the "ultrafine" grid of Gaussian 09, and default SCF convergence criteria were used (RMS of the change in the density matrix < 10^{-8}, maximum change in density matrix < 10^{-6}). Before the geometry optimization step, the wavefunction was checked for internal instabilities. Wavefunctions thus found to be instable were re-optimized to real, spin-restricted or spin-unrestricted wavefunctions, for singlet and quintet states, respectively. Molecular geometries were optimized using tight convergence criteria (maximum force < 0.000015, RMS force < 0.000010, maximum displacement < 0.00006, RMS displacement < 0.00004). All stationary points were characterized by the eigenvalues of the analytically calculated Hessian matrix, and confirmed to be minima (i.e., no imaginary frequencies).

Conformations of $[Fe(tame)_2]^{2+}$

The chelating coordination mode of the tame ligand produces six-membered rings that are classified according to their conformation.[19] The boat conformation corresponds to eclipsed C–N bonds and results in an achiral Fe(tame) fragment. In contrast, skew-boat conformations are chiral and their C–N bonds are non-eclipsed, though not fully staggered. The dihedral angle along the C–N bond is calculated to be 30–33° in gas phase, in models excluding counterions. Such skew-boat conformations can be obtained starting from the three-coordinate Fe(tame) fragment with boat conformation at each six-membered ring (i.e., with all C–N bonds eclipsed) by twisting the tripodal carbon-skeleton along the C_3 axis of the Fe(tame) fragment. The two enantiomeric conformations resulting from twisting clockwise or anti-clockwise are labelled with stereodescriptor δ or λ according to the skew-line convention.[20]

The combination of individual tame ligand conformations in $[Fe(tame)_2]^{2+}$ gives rise to various isomers that were modelled using DFT (B3LYP-D3MBJ/quadruple-ζ; see the Computational Details). Firstly, attempts to model minima with one or two eclipsed (i.e., boat) conformations failed. All these geometry optimizations converged to one of the skew-boat conformers on each tame ligand. Instead, the combinations involving boat conformations are likely transition states connecting skew-boat conformers, at least on the gas-phase B3LYP-D3MBJ potential energy surface of $[Fe(tame)_2]^{2+}$.

The combination of δ or λ skew-boat conformers results in three isomers: δδ, λλ, and δλ.[19] The first two are enantiomers with D_3 symmetry, while δλ is a meso form

with S_6 symmetry. By definition the two enantiomers are associated with identical energy, and the energies calculated for our independently generated δδ and λλ are indeed very similar, with only negligible differences (Table S2). The calculated energy difference between the meso δλ and the enantiomers (δδ or λλ) is slightly larger, but still negligible (below 0.16 kcal/mol). The calculations thus predict that the three conformational isomers may coexist.

Table S2: Calculated energy for conformations and spin states of $[Fe(tame)_2]^{+2}$ in gas phase.

Conformer (*point group*)	Spin State	E [a.u.]	ΔE(wrt lowest) [kcal/mol]
λλ (D_3)	HS	-1991.489238	0.01
λλ (D_3)	LS	-1991.488414	0.53
δλ (S_6)	HS	-1991.489009	0.15
δλ (S_6)	LS	-1991.488167	0.68
δδ (D_3)	HS	-1991.489253	0.00
δδ (D_3)	LS	-1991.488424	0.52

The Structure and LS-HS Energy Gap of the Iron Complex

In the following we analyse the δλ conformer as modelled using different DFT methods. This conformer was chosen because it matches the conformation of the crystal structure (see Figure S1 and also Figure 1 in the main text). In our earlier DFT modelling of $[Fe(tame)_2]^{2+}$,[16c] we exploited the use of an implicit solvent model (e.g., COSMO, PCM) as an approximate, empirically selected representation of the effects of the condensed phase.[16b] This strategy was coupled with the OPBE functional that is considered the functional of choice for reproducing spin-state energies of iron complexes.[16a] This computational protocol gives a low-spin ground state for $[Fe(tame)_2]^{2+}$, albeit by a very small margin ($\Delta E_{HS\text{-}LS}$ = 3.1 kcal/mol, see Table S3), and an average Fe–N bond distance of 2.015 Å for the low-spin and 2.223 Å for the high-spin state.

Both the LFMM-driven *de novo* design that predicted the Fe^{II}-tame complex as a potential spin-crossover centre and the accompanying DFT calculations that corroborated this prediction[16c] were based on the naked $[Fe(tame)_2]^{2+}$ cluster model. Modelling the candidate spin-crossover compounds as naked Fe^{II} cations was chosen in the interest of computational efficiency. *De novo* design runs may involve large numbers of candidate molecules and the simple cluster model thus proved useful in identifying the $[Fe(tame)_2]^{2+}$ fragment as a potential spin-crossover centre. Gratifyingly, this potential is confirmed by the here-reported spin-crossover properties of the corresponding chloride salt. However, at the same time it is clear that the small energy gap ($\Delta E_{HS\text{-}LS}$ = 3.1 kcal/mol) can be overcome by effects external to the $[Fe(tame)_2]^{2+}$ complex. Further crystallographic studies of other salts of $[Fe(tame)_2]^{2+}$ (and in different lattices) will be required to better explore this nexus between theory and experiment.

Table S3: Calculated energy and bond lengths for $[Fe(tame)_2]^{+2}$ and $[Fe(tame)_2]Cl_2$.

Model	Spin state	Computational Method	E [a.u.]	$\overline{r}_{Fe-N}$ [Å]	ΔE_{HS-LS} [kcal/mol]
$[Fe(tame)_2]^{+2}$	HS	OPBE-PCM(water)/cc-pVTZ	-1991.367339	2.223	3.1
$[Fe(tame)_2]^{+2}$	LS	OPBE-PCM(water)/cc-pVTZ	-1991.372205	2.015	
$[Fe(tame)_2]^{+2}$	HS	BP86-D3MBJ/cc-pVTZ	-1991.530933	2.235	17.0
$[Fe(tame)_2]^{+2}$	LS	BP86-D3MBJ/cc-pVTZ	-1991.557956	2.039	
$[Fe(tame)_2]^{+2}$	HS	PBEPBE-D3MBJ/cc-pVTZ	-1990.138753	2.234	12.2
$[Fe(tame)_2]^{+2}$	LS	PBEPBE-D3MBJ/cc-pVTZ	-1990.158147	2.038	
$[Fe(tame)_2]Cl_2$	HS	OPBE-PCM(water)/cc-pVTZ	-2912.109121	2.224	2.9
$[Fe(tame)_2]Cl_2$	LS	OPBE-PCM(water)/cc-pVTZ	-2912.113694	2.012	
$[Fe(tame)_2]Cl_2$	HS	BP86-D3MBJ/cc-pVTZ	-2912.659347	2.211	17.9
$[Fe(tame)_2]Cl_2$	LS	BP86-D3MBJ/cc-pVTZ	-2912.687869	2.030	
$[Fe(tame)_2]Cl_2$	HS	PBEPBE-D3MBJ/cc-pVTZ	-2910.795192	2.212	12.9
$[Fe(tame)_2]Cl_2$	LS	PBEPBE-D3MBJ/cc-pVTZ	-2910.815798	2.029	

Turning now to the Fe−N distances of the chloride salt, the above-described OPBE-PCM-based protocol in combination with the cationic cluster model gives shorter bonds than that observed for the low spin state (2.015 Å *vs.* 2.035 Å) and for the high spin form longer bonds than those found experimentally (2.223 Å *vs.* 2.189 Å) (Tables S1 and S2). In comparison, the dispersion-corrected BP86-D3MBJ and PBE-D3MBJ functionals, which are here used without implicit solvent, predict Fe–N bonds of the low-spin state in better agreement with experiment, but the corresponding bond distances of the high-spin state are still overestimated. While we will discuss the reasons for the discrepancy between the calculated and observed bond distances below, we note in passing that the DFT-D3MBJ computational models that have not been developed specifically to reproduce energy differences between spin states clearly overestimate the stability of the low-spin relative to the high-spin state (ΔE_{HS-LS} is equal to 17.0 and 12.2 for BP86-D3MBJ and PBE-D3MBJ, respectively). Some of this added stability is due to the dispersion term (D3MBJ) not used in conjunction with OPBE.[21]

To analyse the above-mentioned disagreement between the experimental and computed Fe–N bonds distances, we extend the cationic cluster model by including two chloride ions, in anionic positions found in the crystal structure (see Figure S3). While the addition of counterions has negligible effects on ΔE_{HS-LS}, the resulting Fe–N bonds are shorter, by 0.023 Å (HS) and 0.009 Å (LS), than those without counterions. The counterions thus improve the agreement with the Fe–N distances from X-ray crystallography. Nevertheless, the two added chlorides distort the geometry of the complex. The effect is that one of the three C–N bonds of each ligand is twisted toward the corresponding eclipsed conformation (see Figure S3). The

twisting is more pronounced for the high-spin state, for which a fully eclipsed conformation is reached. The twisting is predicted to be less pronounced by the OPBE-PCM computational model in which the implicit polar solvent (water) weakens the bonding interactions between Cl and HN. This leads to longer (by ca. 10%) Cl–Fe distances when the implicit solvation model is used.

The above-described twisting of the C–N bonds is an artefact resulting from having only two chloride anions around the complex, compared to the six ions of the crystal structure. Nevertheless, the chloride-induced shortening of the Fe–N bonds, and in particular the pronounced shortening for the high spin state, is a result of interactions between the chloride and the tame ligands that also exist in the crystal structure. In the present two-chloride models, each chloride is located approximately in the plane of the three N atoms of a single tame ligand. The chloride ion interacts with two of the NH units from that ligand and with one unit from the other tame ligand (Figure S3). As a consequence, the latter NH group (there are two such groups in each $[Fe(tame)_2]Cl_2$ model) is bent towards the chloride. To illustrate, with the PBE-D3MBJ computational model the C–N–H angle is 114° and 106°–110° for the bent and unbent NH groups, respectively (Figure S3). Moreover, among the three NH groups interacting with a single chloride, the bent NH group is the one that is closest to the metal (2.17 Å *vs.* 2.23 Å using PBE-D3MBJ, see Figure S3). Similar shortening of the M–N bonds in $[M(tame)_2]Cl_2$ was previously observed for the corresponding nickel complexes.[19]

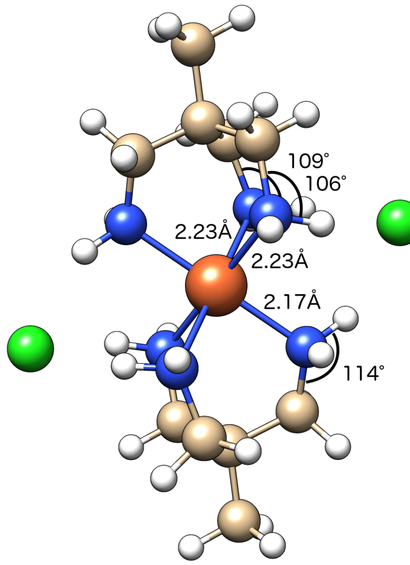

Figure S3: Structure of $[Fe(tame)_2]Cl_2$ as computed with DFT model PBEPBE-D3MBJ/cc-pVTZ. Chloride atoms in green, iron in orange, carbon in beige, nitrogen in blue, and hydrogen in white.

Spin crossover from LS to HS results in elongation along the C_3 axis of the $[Fe(tame)_2]^{2+}$ complex, but this direction is perpendicular to the planes of Cl and NH groups in which the above-described shortening of Fe–N bonds takes place. The

compromise between elongation due to high spin and compression due to cation–anion interaction explains the rather short Fe–N bonds recorded experimentally for the high spin state (i.e. 2.189 Å) as well as the less pronounced shortening of these bonds upon spin pairing (ca. 0.15 Å here *vs.* 0.16–0.21 Å for most other $Fe^{II}N_6$ complexes).[22] Conversely, while transition from the LS to the HS state leads to longer Fe–N bonds, the network of NH–Cl interactions favours the more compact coordination sphere of the low spin state, thereby reducing the magnitude of the Fe–N bonds elongation.

Cartesian coordinates of DFT-optimized geometries

λλ-[Fe(tame)$_2$]$^{+2}$ (HS) B3LYP-GD3BJMod/cc-pVQZ

```
C  +2.64785090E-03 +1.69923008E-02 +3.00405771E-02
N  -1.13655904E+00 -6.93592111E-01 -6.27412590E-01
H  -8.78241492E-01 -1.66752778E+00 -7.46839222E-01
H  -1.91464333E+00 -7.19163115E-01 +2.36454330E-02
C  -3.33349960E-02 +1.54729589E+00 -1.32110953E-01
C  +5.68700861E-01 +1.99132615E+00 -1.47857919E+00
N  +1.73218290E-01 +1.14108446E+00 -2.64196838E+00
H  +3.40776267E-01 +1.67298745E+00 -3.48939617E+00
H  +8.22785027E-01 +3.64394704E-01 -2.70067651E+00
H  +2.65986956E-01 +3.01529928E+00 -1.69349181E+00
H  +1.65421504E+00 +2.00405462E+00 -1.39004656E+00
C  -1.45598264E+00 +2.10045313E+00 +6.19346420E-02
H  -1.39013816E+00 +3.14268826E+00 +3.71153097E-01
H  -1.94790150E+00 +1.56852910E+00 +8.75226583E-01
N  -2.32591939E+00 +1.99271725E+00 -1.14894819E+00
H  -3.29276436E+00 +2.07140442E+00 -8.52000412E-01
H  -2.17020057E+00 +2.81314190E+00 -1.72490586E+00
C  +8.42664246E-01 +2.14552618E+00 +9.78811601E-01
H  +3.87649962E-01 +1.99810401E+00 +1.95721568E+00
H  +9.82006367E-01 +3.21555979E+00 +8.31956610E-01
H  +1.82654605E+00 +1.67886319E+00 +9.93141935E-01
H  +1.67061235E-02 -2.25290912E-01 +1.09174655E+00
H  +9.28015279E-01 -3.75974976E-01 -3.88384810E-01
Fe -1.89529640E+00 +2.51761085E-01 -2.49855242E+00
N  -3.90773309E+00 -7.55790200E-01 -2.33414093E+00
H  -4.52250852E+00 -3.48471901E-01 -1.63752063E+00
H  -3.73782466E+00 -1.69773075E+00 -1.99869713E+00
C  -4.66526902E+00 -8.49345574E-01 -3.61874209E+00
C  -3.78000027E+00 -1.00075080E+00 -4.87008924E+00
C  -3.27143776E+00 +3.61841976E-01 -5.37226785E+00
N  -2.78129825E+00 +1.27009012E+00 -4.29074253E+00
H  -2.18823626E+00 +1.97555734E+00 -4.71474809E+00
H  -3.57421758E+00 +1.78958875E+00 -3.92954506E+00
H  -2.45492738E+00 +2.10638232E-01 -6.07712539E+00
H  -4.06955861E+00 +8.53770776E-01 -5.92651312E+00
C  -2.61829072E+00 -1.98165205E+00 -4.62986356E+00
H  -2.31396378E+00 -2.40882327E+00 -5.58434245E+00
H  -2.95948306E+00 -2.81284387E+00 -4.01462672E+00
N  -1.43137069E+00 -1.36994916E+00 -3.95754834E+00
H  -8.69700603E-01 -2.11648184E+00 -3.56160295E+00
H  -8.36633962E-01 -9.60837435E-01 -4.67089096E+00
C  -4.65843136E+00 -1.59561570E+00 -5.98091543E+00
H  -4.92869584E+00 -2.62595287E+00 -5.75427457E+00
H  -4.13726020E+00 -1.58855817E+00 -6.93714036E+00
H  -5.57928107E+00 -1.02595761E+00 -6.09706385E+00
H  -5.35495833E+00 -1.69113941E+00 -3.57347838E+00
H  -5.27875539E+00 +4.57791342E-02 -3.71208006E+00
```

λλ-[Fe(tame)$_2$]$^{+2}$ (LS) B3LYP-GD3BJMod/cc-pVQZ

```
C  -4.55062376E-02 -2.48641728E-02 -7.46534006E-02
N  -1.17811004E+00 -7.06242282E-01 -7.74588111E-01
H  -9.11504685E-01 -1.67262463E+00 -9.33334932E-01
```

H -1.95321989E+00 -7.66628963E-01 -1.22571105E-01
C -8.92621548E-02 +1.50574341E+00 -2.02687147E-01
C +4.82009531E-01 +1.95807316E+00 -1.55540351E+00
N +2.36231494E-02 +1.11165675E+00 -2.70016354E+00
H +1.50331077E-01 +1.64541544E+00 -3.55410204E+00
H +6.73516838E-01 +3.37913118E-01 -2.79248686E+00
H +1.89546399E-01 +2.98767430E+00 -1.75635889E+00
H +1.56997842E+00 +1.94721816E+00 -1.50883257E+00
C -1.52082097E+00 +2.02976263E+00 -1.08730611E-02
H -1.48402528E+00 +3.07270217E+00 +3.00368178E-01
H -2.01185784E+00 +1.48263150E+00 +7.92584176E-01
N -2.36270845E+00 +1.90615164E+00 -1.24109851E+00
H -3.33677375E+00 +1.94409305E+00 -9.58430846E-01
H -2.23120126E+00 +2.74816264E+00 -1.79182085E+00
C +7.87371974E-01 +2.10063471E+00 +9.06344270E-01
H +3.45668592E-01 +1.92982647E+00 +1.88703417E+00
H +9.06691346E-01 +3.17520143E+00 +7.75620328E-01
H +1.77928396E+00 +1.65103167E+00 +9.03240719E-01
H -5.28768818E-02 -2.98632680E-01 +9.79374337E-01
H +8.87123103E-01 -4.05688847E-01 -4.88382548E-01
Fe -1.90546034E+00 +2.71353453E-01 -2.50053779E+00
N -3.79921258E+00 -6.45911100E-01 -2.28967077E+00
H -4.39139286E+00 -2.06037044E-01 -1.59254466E+00
H -3.63554188E+00 -1.57797889E+00 -1.92355899E+00
C -4.59463921E+00 -7.83792936E-01 -3.54876033E+00
C -3.72054795E+00 -9.64518581E-01 -4.79933547E+00
C -3.18406532E+00 +3.88354820E-01 -5.29182747E+00
N -2.70464606E+00 +1.27049813E+00 -4.18310587E+00
H -2.07899311E+00 +1.96260824E+00 -4.58273201E+00
H -3.49684749E+00 +1.81273936E+00 -3.85452695E+00
H -2.35634969E+00 +2.30545520E-01 -5.98175454E+00
H -3.96318234E+00 +9.02047686E-01 -5.85311094E+00
C -2.56805621E+00 -1.94283542E+00 -4.52456159E+00
H -2.22528177E+00 -2.36922076E+00 -5.46619286E+00
H -2.92350049E+00 -2.77440041E+00 -3.91780378E+00
N -1.41398047E+00 -1.30860854E+00 -3.81528348E+00
H -8.69651327E-01 -2.04718153E+00 -3.38126716E+00
H -7.92626529E-01 -9.23136320E-01 -4.51876614E+00
C -4.59586573E+00 -1.56083931E+00 -5.90868996E+00
H -4.88656464E+00 -2.58281580E+00 -5.66974481E+00
H -4.06414547E+00 -1.57770267E+00 -6.85896152E+00
H -5.50532590E+00 -9.77330063E-01 -6.04447700E+00
H -5.27340771E+00 -1.63057419E+00 -3.45825246E+00
H -5.21689184E+00 +1.03525694E-01 -3.65571178E+00

δλ-$[Fe(tame)_2]^{+2}$ (HS) B3LYP-GD3BJMod/cc-pVQZ

Fe -1.44568000E-05 -6.94950000E-06 +2.05880000E-06
N +1.96266023E-01 +1.71842484E+00 -1.41275823E+00
H -2.69943269E-01 +2.56098628E+00 -1.09297382E+00
C -2.44664207E-01 +1.44453470E+00 -2.81493378E+00
C -9.07061200E-04 -2.47099590E-03 -3.27957229E+00
C -4.64777900E-03 +3.41219830E-03 -4.81560118E+00
H +1.32290724E-02 -1.01169433E+00 -5.20957494E+00
H +8.64895085E-01 +5.29833669E-01 -5.20651077E+00
H -8.95954929E-01 +4.96244001E-01 -5.20119547E+00
C -1.13038899E+00 -9.44651247E-01 -2.82269553E+00
N -1.59919972E+00 -7.11941473E-01 -1.42322840E+00
H -2.09347396E+00 -1.54364131E+00 -1.11786685E+00
H -2.31181804E+00 +9.25291270E-03 -1.44489256E+00
H -1.97025799E+00 -8.43858848E-01 -3.50887871E+00
H -7.95709743E-01 -1.97852901E+00 -2.89633868E+00
C +1.37902697E+00 -5.12789946E-01 -2.82952878E+00
N +1.42225985E+00 -9.99332877E-01 -1.41653444E+00
H +2.39046823E+00 -1.00286913E+00 -1.11347466E+00
H +1.15175549E+00 -1.97693232E+00 -1.40959229E+00
H +1.69542690E+00 -1.31144022E+00 -3.49901867E+00
H +2.11488975E+00 +2.83772341E-01 -2.93114132E+00
H +2.66731419E-01 +2.12438692E+00 -3.49494718E+00
H -1.30567295E+00 +1.67975596E+00 -2.88373274E+00

H +1.17946408E+00 +1.96883818E+00 -1.43195657E+00
N +1.59917549E+00 +7.11964318E-01 +1.42320695E+00
H +2.31180718E+00 -9.21665700E-03 +1.44487908E+00
C +1.13036581E+00 +9.44688104E-01 +2.82267129E+00
C +9.17382000E-04 +2.47900120E-03 +3.27957203E+00
C +4.68180840E-03 -3.39408140E-03 +4.81560072E+00
H +8.96012343E-01 -4.96192271E-01 +5.20118396E+00
H -8.64836531E-01 -5.29842968E-01 +5.20652807E+00
H -1.32236140E-02 +1.01171451E+00 +5.20956773E+00
C +2.44706054E-01 -1.44452319E+00 +2.81493728E+00
N -1.96250034E-01 -1.71843647E+00 +1.41277388E+00
H -1.17944196E+00 -1.96887233E+00 +1.43199606E+00
H +2.69971574E-01 -2.56099039E+00 +1.09298771E+00
H +1.30572334E+00 -1.67971331E+00 +2.88371174E+00
H -2.66652519E-01 -2.12438604E+00 +3.49496807E+00
C -1.37903695E+00 +5.12757150E-01 +2.82954620E+00
N -1.42230862E+00 +9.99278199E-01 +1.41654467E+00
H -1.15185526E+00 +1.97689156E+00 +1.40958499E+00
H -2.39052031E+00 +1.00276016E+00 +1.11349473E+00
H -2.11487818E+00 -2.83821689E-01 +2.93118513E+00
H -1.69544423E+00 +1.31140992E+00 +3.49902941E+00
H +7.95652121E-01 +1.97855606E+00 +2.89629372E+00
H +1.97024196E+00 +8.43938705E-01 +3.50885185E+00
H +2.09343337E+00 +1.54366837E+00 +1.11783049E+00

δλ-$[Fe(tame)_2]^{+2}$ (LS) B3LYP-GD3BJMod/cc-pVQZ
C -2.48156749E-02 +2.99889976E-02 -8.82324278E-02
N +2.64626548E-01 -3.48722189E-01 -1.50599967E+00
H +1.05009467E+00 -9.90809690E-01 -1.49931382E+00
H -5.11568299E-01 -9.09709338E-01 -1.84238953E+00
C +7.83292842E-01 +1.24639151E+00 +3.88706840E-01
C +2.24095904E+00 +1.15503467E+00 -8.79512972E-02
H +2.59656873E+00 +1.29281052E-01 -2.30799940E-03
H +2.87163446E+00 +1.75819354E+00 +5.63509936E-01
N +2.42453664E+00 +1.59482336E+00 -1.50574807E+00
H +3.29832074E+00 +1.20285091E+00 -1.84215923E+00
H +2.58784884E+00 +2.59611386E+00 -1.49948690E+00
C +1.33609688E-01 +2.55451955E+00 -8.77649840E-02
N -3.38845789E-01 +2.49383549E+00 -1.50568266E+00
H -1.28779304E+00 +2.13505488E+00 -1.49950095E+00
H -4.35929703E-01 +3.44661798E+00 -1.84197689E+00
H +8.44086746E-01 +3.37537402E+00 -1.88148950E-03
H -7.04199555E-01 +2.79889676E+00 +5.63619551E-01
C +7.83105567E-01 +1.24609949E+00 +1.92240463E+00
H -2.30540815E-01 +1.16656373E+00 +2.31258129E+00
H +1.22101231E+00 +2.16350842E+00 +2.31307244E+00
H +1.35858132E+00 +4.07941894E-01 +2.31272650E+00
H +1.81763903E-01 -8.17938192E-01 +5.63155742E-01
H -1.09092517E+00 +2.35226305E-01 -2.90396550E-03
Fe +7.83138321E-01 +1.24629995E+00 -2.79135107E+00
N -8.58260000E-01 +8.97776544E-01 -4.07695405E+00
H -1.73204412E+00 +1.28974892E+00 -3.74054283E+00
H -1.02157216E+00 -1.03513957E-01 -4.08321530E+00
C -6.74682444E-01 +1.33756539E+00 -5.49475077E+00
C +7.82983734E-01 +1.24620853E+00 -5.97140897E+00
C +1.59109231E+00 +2.46261100E+00 -5.49446967E+00
N +1.30165014E+00 +2.84132211E+00 -4.07670240E+00
H +2.07784502E+00 +3.40230920E+00 -3.74031252E+00
H +5.16182051E-01 +3.48340966E+00 -4.08338819E+00
H +2.65720179E+00 +2.25737365E+00 -5.57979817E+00
H +1.38451275E+00 +3.31053823E+00 -6.14585779E+00
C +1.43266687E+00 -6.19195444E-02 -5.49493723E+00
H +2.27047610E+00 -3.06296728E-01 -6.14632180E+00
H +7.22189799E-01 -8.82773995E-01 -5.58082077E+00
N +1.90512238E+00 -1.23558740E-03 -4.07701956E+00
H +2.00220624E+00 -9.54018103E-01 -3.74072539E+00
H +2.85406965E+00 +3.57544962E-01 -4.08320126E+00
C +7.83170938E-01 +1.24650065E+00 -7.50510676E+00
H +3.45264160E-01 +3.29091749E-01 -7.89577461E+00

```
H  +1.79681730E+00 +1.32603642E+00 -7.89528347E+00
H  +2.07695178E-01 +2.08465828E+00 -7.89542855E+00
H  -1.30535791E+00 +7.34406622E-01 -6.14621205E+00
H  -1.03029210E+00 +2.36331904E+00 -5.58039393E+00
```

δδ-$[Fe(tame)_2]^{+2}$ (HS) B3LYP-GD3BJMod/cc-pVQZ

```
C  +3.06847940E-03 -5.36394305E-02 -5.84027681E-02
N  -2.18763735E-01 +3.76451774E-01 +1.35595219E+00
H  -3.45869872E-01 +1.38284293E+00 +1.36472065E+00
H  -1.11167644E+00 +5.57130740E-03 +1.66307441E+00
C  +1.47044797E+00 +1.69600271E-02 -5.16289973E-01
C  +2.14454442E+00 +1.32417823E+00 -5.85755239E-02
H  +1.43233482E+00 +2.14537486E+00 -1.27443695E-01
H  +2.95417279E+00 +1.56105190E+00 -7.47589997E-01
N  +2.67287727E+00 +1.28972043E+00 +1.33872189E+00
H  +2.79808551E+00 +2.24724495E+00 +1.64951745E+00
H  +3.61284375E+00 +9.09796121E-01 +1.31151648E+00
C  +2.26266558E+00 -1.22147378E+00 -6.00652632E-02
N  +1.97068062E+00 -1.65073135E+00 +1.34192748E+00
H  +1.16494045E+00 -2.26752340E+00 +1.32509477E+00
H  +2.73179948E+00 -2.24200065E+00 +1.65919309E+00
H  +3.33084301E+00 -1.02282307E+00 -1.33397066E-01
H  +2.05433417E+00 -2.04466571E+00 -7.42027202E-01
C  +1.46814504E+00 +1.83267295E-02 -2.05228034E+00
H  +8.71603082E-01 -8.04842156E-01 -2.44288063E+00
H  +2.47915357E+00 -8.54210616E-02 -2.44356277E+00
H  +1.05266650E+00 +9.46985529E-01 -2.44079242E+00
H  -6.04109310E-01 +5.58082235E-01 -7.24236334E-01
H  -3.63201111E-01 -1.07450468E+00 -1.58596507E-01
Fe +1.48876978E+00 +6.73240000E-06 +2.76178181E+00
N  +4.04497008E-01 -1.37583333E+00 +4.18381611E+00
H  -5.20720371E-01 -1.65241075E+00 +3.87298563E+00
C  +2.86178502E-01 -8.60526081E-01 +5.58135724E+00
C  +1.46840723E+00 +1.41840643E-02 +6.03932953E+00
C  +1.46646437E+00 +1.55134564E-02 +7.57539430E+00
H  +4.83061629E-01 +2.76219054E-01 +7.96388576E+00
H  +2.18305628E+00 +7.36343871E-01 +7.96646077E+00
H  +1.73116656E+00 -9.65878447E-01 +7.96631080E+00
C  +1.30308941E+00 +1.47411414E+00 +5.58248476E+00
N  +8.44979406E-01 +1.62589214E+00 +4.16760873E+00
H  +1.07047573E+00 +2.56636892E+00 +3.86126591E+00
H  -1.68845621E-01 +1.59250586E+00 +4.15744444E+00
H  +6.00965250E-01 +1.97452694E+00 +6.24791170E+00
H  +2.25181098E+00 +1.99933077E+00 +5.68463117E+00
C  +2.81791466E+00 -5.69271287E-01 +5.58334466E+00
N  +3.19534125E+00 -2.12345077E-01 +4.18143378E+00
H  +3.90114620E+00 -8.68870901E-01 +3.86461183E+00
H  +3.67506054E+00 +6.81820058E-01 +4.19831917E+00
H  +3.59693286E+00 -2.31789464E-01 +6.26550111E+00
H  +2.79294946E+00 -1.65546262E+00 +5.65659513E+00
H  +1.82468891E-01 -1.69801322E+00 +6.26997353E+00
H  -6.38602800E-01 -2.89311382E-01 +5.65087233E+00
H  +9.29846787E-01 -2.24297522E+00 +4.21040178E+00
```

δδ-$[Fe(tame)_2]^{+2}$ (LS) B3LYP-GD3BJMod/cc-pVQZ

```
C  -1.97867982E-02 +1.91925547E-02 +8.53976170E-02
N  +1.05690296E-01 -2.12892192E-01 +1.55769743E+00
H  +3.84431916E-01 -1.17868479E+00 +1.69598255E+00
H  +8.98973901E-01 +3.24620150E-01 +1.89226982E+00
C  -1.41701751E+00 -3.11444935E-01 -4.61764565E-01
C  -1.94524099E+00 -1.62116766E+00 +1.43403790E-01
H  -1.13985930E+00 -2.35128546E+00 +2.08838030E-01
H  -2.69750750E+00 -2.04656651E+00 -5.19235188E-01
N  -2.52689820E+00 -1.44544119E+00 +1.51003140E+00
H  -2.53674746E+00 -2.35286670E+00 +1.96460452E+00
H  -3.50829685E+00 -1.21198939E+00 +1.40090470E+00
C  -2.39415994E+00 +8.45236495E-01 -2.00901201E-01
N  -2.26062531E+00 +1.43026933E+00 +1.16909595E+00
H  -1.55578845E+00 +2.15897661E+00 +1.12692199E+00
```

```
H  -3.11939505E+00 +1.92775100E+00 +1.38165724E+00
H  -3.41973011E+00 +5.00170711E-01 -3.23921282E-01
H  -2.23685996E+00 +1.62449929E+00 -9.45203969E-01
C  -1.30027517E+00 -5.02143672E-01 -1.97911923E+00
H  -7.96825889E-01 +3.45626549E-01 -2.44156413E+00
H  -2.28357371E+00 -5.98010405E-01 -2.43733506E+00
H  -7.31257437E-01 -1.39969908E+00 -2.21712480E+00
H  +7.23678128E-01 -5.77817709E-01 -4.40720708E-01
H  +2.20401469E-01 +1.06229279E+00 -1.15232528E-01
Fe -1.65922137E+00 +8.37112957E-02 +2.68300932E+00
C  -7.85404706E-01 +1.40140092E+00 +5.31703204E+00
N  -8.61002173E-01 +1.65848662E+00 +3.84553022E+00
H  -1.46163571E+00 +2.46369288E+00 +3.70257330E+00
H  +5.30546042E-02 +1.97246813E+00 +3.53561388E+00
C  -1.90248186E+00 +4.78175344E-01 +5.82746883E+00
C  -3.25209454E+00 +8.51239247E-01 +5.19498279E+00
H  -3.34934829E+00 +1.93449962E+00 +5.13937618E+00
H  -4.05965599E+00 +4.98931479E-01 +5.83505643E+00
N  -3.42970477E+00 +2.96228343E-01 +3.81743038E+00
H  -4.15055169E+00 +8.38646617E-01 +3.35235184E+00
H  -3.83478704E+00 -6.29771463E-01 +3.90684030E+00
C  -1.56047284E+00 -9.95173248E-01 +5.55746720E+00
N  -9.80477142E-01 -1.22449584E+00 +4.19811162E+00
H  +2.43009354E-02 -1.10014087E+00 +4.26546967E+00
H  -1.09431254E+00 -2.20721551E+00 +3.97129126E+00
H  -2.45597274E+00 -1.60766975E+00 +5.65194707E+00
H  -8.61396404E-01 -1.34741639E+00 +6.31455634E+00
C  -2.02004800E+00 +6.68501458E-01 +7.34483432E+00
H  -1.05103925E+00 +5.54966186E-01 +7.82873792E+00
H  -2.69869506E+00 -6.44059673E-02 +7.77878987E+00
H  -2.40014737E+00 +1.66025306E+00 +7.58566102E+00
H  -8.27233334E-01 +2.34818803E+00 +5.85350383E+00
H  +1.87297198E-01 +9.62974049E-01 +5.53528200E+00
```

$[Fe(tame)_2]Cl_2$ (HS) OPBE-PCM(water)/cc-pVTZ

```
Cl +9.67759617E-01 +6.89207886E+00 -1.18715463E+00
Cl +6.90799623E+00 +2.77825544E+00 -5.52481339E+00
Fe +3.90144290E+00 +4.67843794E+00 -3.27852246E+00
N  +1.72742743E+00 +4.42501231E+00 -3.39124518E+00
H  +1.42922390E+00 +4.99569216E+00 -4.17736347E+00
H  +1.25757175E+00 +4.84061473E+00 -2.58447236E+00
N  +3.53803005E+00 +6.87622747E+00 -3.57143822E+00
H  +2.62861115E+00 +7.00719549E+00 -3.12431529E+00
H  +3.41274470E+00 +7.12332057E+00 -4.54664028E+00
N  +3.77162558E+00 +4.01694952E+00 -5.40871955E+00
H  +4.69974587E+00 +3.60936739E+00 -5.57022091E+00
C  +2.73194567E+00 +3.01972301E+00 -5.73140585E+00
C  +2.19052919E+00 +2.21370374E+00 -4.53453683E+00
H  +3.10931301E+00 +2.30181946E+00 -6.47230178E+00
H  +1.88994334E+00 +3.53545254E+00 -6.20595790E+00
C  +3.31509263E+00 +1.54427430E+00 -3.72797849E+00
C  +1.26919287E+00 +3.05007461E+00 -3.62313188E+00
H  +3.86574453E+00 +8.72869912E-01 -4.39719689E+00
H  +2.84102531E+00 +9.14560714E-01 -2.96778027E+00
H  +1.19043352E+00 +2.54852821E+00 -2.65272705E+00
H  +2.61037016E-01 +3.05550850E+00 -4.05898461E+00
C  +1.33547680E+00 +1.07885162E+00 -5.10882929E+00
H  +8.72577917E-01 +4.94261814E-01 -4.30707059E+00
H  +1.93977397E+00 +3.99625717E-01 -5.71857430E+00
H  +5.34893380E-01 +1.48159192E+00 -5.73751465E+00
H  +3.69947627E+00 +4.78957447E+00 -6.06185000E+00
N  +3.98912168E+00 +5.42022030E+00 -1.17326597E+00
H  +4.02796182E+00 +4.69353850E+00 -4.66196104E-01
C  +5.04262945E+00 +6.41303029E+00 -8.79380963E-01
C  +5.60055658E+00 +7.17189565E+00 -2.09916916E+00
H  +5.87500910E+00 +5.89770238E+00 -3.87290105E-01
H  +4.67551911E+00 +7.15797916E+00 -1.60027378E-01
C  +6.52745845E+00 +6.29512897E+00 -2.96649167E+00
C  +4.48876927E+00 +7.81560403E+00 -2.94318633E+00
```

```
H  +7.52915278E+00 +6.29992458E+00 -2.51536225E+00
H  +6.62458564E+00 +6.76012341E+00 -3.95337701E+00
H  +4.97439646E+00 +8.40611409E+00 -3.72739158E+00
H  +3.93858813E+00 +8.52098967E+00 -2.30905666E+00
C  +6.45637326E+00 +8.32257826E+00 -1.55868292E+00
H  +6.92798906E+00 +8.87734222E+00 -2.37654213E+00
H  +7.24971961E+00 +7.93937090E+00 -9.08711891E-01
H  +5.84970921E+00 +9.02496373E+00 -9.78646665E-01
H  +3.06726579E+00 +5.86019308E+00 -1.05743417E+00
N  +4.26116929E+00 +2.46283439E+00 -3.06093246E+00
H  +5.17204936E+00 +2.35373178E+00 -3.50649445E+00
H  +4.38678724E+00 +2.17063857E+00 -2.09860460E+00
N  +6.06515065E+00 +4.91570283E+00 -3.15617371E+00
H  +6.53098040E+00 +4.47357734E+00 -3.95771648E+00
H  +6.35782299E+00 +4.36714370E+00 -2.35261923E+00
```

$[Fe(tame)_2]Cl_2$ (LS) OPBE-PCM(water)/cc-pVTZ

```
Cl +4.34485851E+00 +6.81037302E+00 +1.52568724E+00
Cl +4.16172905E+00 +3.78209763E+00 -6.36828431E+00
Fe +4.25299625E+00 +5.29683695E+00 -2.42133291E+00
N  +4.36444498E+00 +4.30367971E+00 -6.82357095E-01
H  +3.41533421E+00 +4.03008812E+00 -4.32107275E-01
H  +4.64422278E+00 +4.91247066E+00 +9.56028343E-02
N  +2.57406088E+00 +6.18044488E+00 -1.74361297E+00
H  +2.43418443E+00 +5.92956339E+00 -7.66156651E-01
H  +1.76343235E+00 +5.80653284E+00 -2.23099385E+00
N  +3.26230714E+00 +3.70010973E+00 -3.15131318E+00
H  +3.46600838E+00 +3.65248923E+00 -4.16062905E+00
C  +3.60776287E+00 +2.38851087E+00 -2.55598915E+00
C  +5.04712705E+00 +2.32550550E+00 -2.02190311E+00
H  +3.48004541E+00 +1.59841618E+00 -3.30661428E+00
H  +2.90403482E+00 +2.16425818E+00 -1.74669353E+00
C  +6.00646948E+00 +2.93195535E+00 -3.04624672E+00
C  +5.17052795E+00 +3.07499807E+00 -6.86040583E-01
H  +5.76417291E+00 +2.54840615E+00 -4.04170941E+00
H  +7.03038425E+00 +2.61995639E+00 -2.80842479E+00
H  +6.21783179E+00 +3.34465755E+00 -5.11922252E-01
H  +4.86813612E+00 +2.41295220E+00 +1.36805134E-01
C  +5.42747531E+00 +8.60073034E-01 -1.81469329E+00
H  +6.41993939E+00 +7.74805422E-01 -1.35953443E+00
H  +5.44643404E+00 +3.22167137E-01 -2.76828078E+00
H  +4.70872619E+00 +3.61096331E-01 -1.15686063E+00
H  +2.24953892E+00 +3.79818081E+00 -3.10127570E+00
N  +5.24361881E+00 +6.89359496E+00 -1.69128447E+00
H  +6.25639970E+00 +6.79554146E+00 -1.74110959E+00
C  +4.89823374E+00 +8.20516103E+00 -2.28673420E+00
C  +3.45881555E+00 +8.26814474E+00 -2.82073388E+00
H  +5.60193285E+00 +8.42929788E+00 -3.09608700E+00
H  +5.02601656E+00 +8.99533672E+00 -1.53620345E+00
C  +3.33533116E+00 +7.51862094E+00 -4.15655358E+00
C  +2.49961824E+00 +7.66173486E+00 -1.79622397E+00
H  +3.63759914E+00 +8.18070071E+00 -4.97941985E+00
H  +2.28803605E+00 +7.24886134E+00 -4.33062676E+00
H  +1.47567747E+00 +7.97390520E+00 -2.03369140E+00
H  +2.74225754E+00 +8.04510315E+00 -8.00765219E-01
C  +3.07841517E+00 +9.73355806E+00 -3.02799055E+00
H  +2.08596666E+00 +9.81876173E+00 -3.48319419E+00
H  +3.79716884E+00 +1.02325492E+01 -3.68580730E+00
H  +3.05939191E+00 +1.02714881E+01 -2.07441803E+00
H  +5.03976820E+00 +6.94123254E+00 -6.81979450E-01
N  +5.93194269E+00 +4.41322696E+00 -3.09904128E+00
H  +6.07193302E+00 +4.66410043E+00 -4.07650288E+00
H  +6.74246755E+00 +4.78721057E+00 -2.61154269E+00
N  +4.14153661E+00 +6.29003084E+00 -4.16029716E+00
H  +3.86176312E+00 +5.68131839E+00 -4.93825231E+00
H  +5.09062399E+00 +6.56369480E+00 -4.41056007E+00
```

$[Fe(tame)_2]Cl_2$ (HS) BP86-GD3BJMod/cc-pVTZ

```
Cl +1.20224064E+00 +6.83574680E+00 -1.46563661E+00
```

```
Cl +6.58989764E+00 +2.55873724E+00 -5.15743552E+00
Fe +3.89607382E+00 +4.69721613E+00 -3.31150091E+00
N  +1.74468208E+00 +4.46096285E+00 -3.43014109E+00
H  +1.45332333E+00 +5.00689437E+00 -4.24550428E+00
H  +1.29804387E+00 +4.95357991E+00 -2.62789080E+00
N  +3.52901257E+00 +6.88452904E+00 -3.57040671E+00
H  +2.60270130E+00 +6.95006078E+00 -3.08427779E+00
H  +3.38994835E+00 +7.20007421E+00 -4.53067491E+00
N  +3.79695227E+00 +3.99786177E+00 -5.43021254E+00
H  +4.72402215E+00 +3.50791282E+00 -5.49029007E+00
C  +2.71252587E+00 +3.03252617E+00 -5.74886959E+00
C  +2.19705716E+00 +2.21945073E+00 -4.53746120E+00
H  +3.05255163E+00 +2.31648969E+00 -6.51602602E+00
H  +1.86345383E+00 +3.58267773E+00 -6.18568535E+00
C  +3.35875908E+00 +1.56383300E+00 -3.76229981E+00
C  +1.29550938E+00 +3.06531741E+00 -3.60033282E+00
H  +3.95987302E+00 +9.68843013E-01 -4.46844659E+00
H  +2.91852546E+00 +8.65592205E-01 -3.03486105E+00
H  +1.27555474E+00 +2.59553213E+00 -2.60601325E+00
H  +2.61967345E-01 +3.03841744E+00 -3.98559095E+00
C  +1.33526381E+00 +1.07159362E+00 -5.09014935E+00
H  +8.45918375E-01 +5.21544083E-01 -4.27312718E+00
H  +1.94632072E+00 +3.58693491E-01 -5.66119434E+00
H  +5.48262404E-01 +1.45670800E+00 -5.75451676E+00
H  +3.82817676E+00 +4.71311475E+00 -6.15958760E+00
N  +3.99516499E+00 +5.39657387E+00 -1.19279953E+00
H  +3.96388770E+00 +4.68132518E+00 -4.63422244E-01
C  +5.07962216E+00 +6.36186367E+00 -8.74116436E-01
C  +5.59508635E+00 +7.17497747E+00 -2.08550111E+00
H  +5.92868473E+00 +5.81166940E+00 -4.37335595E-01
H  +4.73962794E+00 +7.07787799E+00 -1.06925023E-01
C  +6.49662639E+00 +6.32914333E+00 -3.02266602E+00
C  +4.43336935E+00 +7.83060710E+00 -2.86062835E+00
H  +7.53017713E+00 +6.35605115E+00 -2.63743174E+00
H  +6.51655062E+00 +6.79894610E+00 -4.01697786E+00
H  +4.87357793E+00 +8.52894278E+00 -3.58799133E+00
H  +3.83220164E+00 +8.42549320E+00 -2.15444106E+00
C  +6.45687626E+00 +8.32282156E+00 -1.53278221E+00
H  +6.94622415E+00 +8.87289113E+00 -2.34978930E+00
H  +7.24387518E+00 +7.93769279E+00 -8.68419962E-01
H  +5.84581552E+00 +9.03570732E+00 -9.61723151E-01
H  +3.06811197E+00 +5.88656397E+00 -1.13275303E+00
N  +4.26318119E+00 +2.50992306E+00 -3.05262390E+00
H  +5.18944813E+00 +2.44440621E+00 -3.53884942E+00
H  +4.40235715E+00 +2.19437097E+00 -2.09237356E+00
N  +6.04746827E+00 +4.93349455E+00 -3.19287232E+00
H  +6.49411244E+00 +4.44088967E+00 -3.99512905E+00
H  +6.33883513E+00 +4.38755817E+00 -2.37751478E+00
```

[Fe(tame)$_2$]Cl$_2$ (LS) BP86-GD3BJMod/cc-pVTZ

```
Cl +4.04543179E+00 +6.74523534E+00 +1.18733119E+00
Cl +4.46053062E+00 +3.84839902E+00 -6.02997133E+00
Fe +4.25300814E+00 +5.29680939E+00 -2.42130884E+00
N  +4.37706518E+00 +4.28330798E+00 -6.75242080E-01
H  +3.41271028E+00 +4.02886234E+00 -4.33020953E-01
H  +4.63696738E+00 +4.91544774E+00 +1.11660390E-01
N  +2.63955103E+00 +6.22609659E+00 -1.58814521E+00
H  +2.81215670E+00 +6.14184938E+00 -5.61537414E-01
H  +1.75208769E+00 +5.75716094E+00 -1.79136260E+00
N  +3.24205318E+00 +3.69850746E+00 -3.15043311E+00
H  +3.49896365E+00 +3.67675945E+00 -4.16999884E+00
C  +3.60439762E+00 +2.36658299E+00 -2.57647519E+00
C  +5.04922498E+00 +2.29904321E+00 -2.03350120E+00
H  +3.48578140E+00 +1.59203986E+00 -3.35090330E+00
H  +2.90528492E+00 +2.11899891E+00 -1.76206524E+00
C  +6.04010468E+00 +2.89407555E+00 -3.04274162E+00
C  +5.16838774E+00 +3.03657421E+00 -6.84699246E-01
H  +5.91007444E+00 +2.40247453E+00 -4.01874780E+00
H  +7.06118592E+00 +2.68644700E+00 -2.68895152E+00
```

```
H  +6.22201852E+00 +3.29160535E+00 -4.98112667E-01
H  +4.84682139E+00 +2.36903598E+00 +1.33134917E-01
C  +5.41709590E+00 +8.23516659E-01 -1.82453267E+00
H  +6.39918939E+00 +7.30325380E-01 -1.33851687E+00
H  +5.46002379E+00 +2.88173006E-01 -2.78333487E+00
H  +4.67692026E+00 +3.21685231E-01 -1.18452015E+00
H  +2.21971763E+00 +3.78341952E+00 -3.13378149E+00
N  +5.26396324E+00 +6.89512169E+00 -1.69218739E+00
H  +6.28629910E+00 +6.81023004E+00 -1.70888289E+00
C  +4.90157338E+00 +8.22705462E+00 -2.26610439E+00
C  +3.45675800E+00 +8.29456777E+00 -2.80911722E+00
H  +5.60069621E+00 +8.47469442E+00 -3.08048832E+00
H  +5.02014072E+00 +9.00157154E+00 -1.49164312E+00
C  +3.33765016E+00 +7.55705429E+00 -4.15793056E+00
C  +2.46585565E+00 +7.69950776E+00 -1.79991523E+00
H  +3.65924964E+00 +8.22460219E+00 -4.97574378E+00
H  +2.28402638E+00 +7.30202725E+00 -4.34456403E+00
H  +1.44478303E+00 +7.90708017E+00 -2.15376416E+00
H  +2.59581206E+00 +8.19113958E+00 -8.23919292E-01
C  +3.08886296E+00 +9.77008931E+00 -3.01808186E+00
H  +2.10677788E+00 +9.86326504E+00 -3.50411785E+00
H  +3.82904216E+00 +1.02719403E+01 -3.65807479E+00
H  +3.04590410E+00 +1.03054245E+01 -2.05927636E+00
H  +5.00709096E+00 +6.91683332E+00 -6.72617024E-01
N  +5.86646593E+00 +4.36750401E+00 -3.25446792E+00
H  +5.69388095E+00 +4.45178742E+00 -4.28107389E+00
H  +6.75393953E+00 +4.83640464E+00 -3.05121695E+00
N  +4.12897159E+00 +6.31032045E+00 -4.16737430E+00
H  +3.86907172E+00 +5.67818531E+00 -4.95427747E+00
H  +5.09333041E+00 +6.56476139E+00 -4.40958349E+00
```

$[Fe(tame)_2]Cl_2$ (HS) PBEPBE-GD3BJMod/cc-pVTZ

```
Cl +1.21691733E+00 +6.82765546E+00 -1.47090212E+00
Cl +6.57522412E+00 +2.56675219E+00 -5.15205456E+00
Fe +3.89606955E+00 +4.69722085E+00 -3.31149830E+00
N  +1.74205892E+00 +4.46069092E+00 -3.42944672E+00
H  +1.44686985E+00 +5.00532148E+00 -4.24342251E+00
H  +1.29749854E+00 +4.95190274E+00 -2.62645373E+00
N  +3.53062211E+00 +6.88441996E+00 -3.57381010E+00
H  +2.60256513E+00 +6.94876329E+00 -3.09282454E+00
H  +3.39772252E+00 +7.19995205E+00 -4.53437918E+00
N  +3.79387036E+00 +4.00277805E+00 -5.43065706E+00
H  +4.72093057E+00 +3.51456922E+00 -5.49256603E+00
C  +2.71129786E+00 +3.03991633E+00 -5.74509250E+00
C  +2.19994846E+00 +2.22434852E+00 -4.53569248E+00
H  +3.04666055E+00 +2.32518573E+00 -6.51600004E+00
H  +1.86017402E+00 +3.58960672E+00 -6.17895123E+00
C  +3.36210895E+00 +1.56915132E+00 -3.76394601E+00
C  +1.30033004E+00 +3.06627895E+00 -3.59639450E+00
H  +3.96490527E+00 +9.78005902E-01 -4.47216814E+00
H  +2.92230981E+00 +8.64665245E-01 -3.04193665E+00
H  +1.28652523E+00 +2.59698245E+00 -2.60149787E+00
H  +2.64461381E-01 +3.03226963E+00 -3.97560096E+00
C  +1.34004996E+00 +1.07776735E+00 -5.08880299E+00
H  +8.52130358E-01 +5.24681433E-01 -4.27309059E+00
H  +1.95014442E+00 +3.66144313E-01 -5.66234166E+00
H  +5.51086595E-01 +1.46180880E+00 -5.75133632E+00
H  +3.82246655E+00 +4.71888337E+00 -6.15854159E+00
N  +3.99828436E+00 +5.39164749E+00 -1.19233549E+00
H  +3.96969376E+00 +4.67553519E+00 -4.64457573E-01
C  +5.08085665E+00 +6.35450828E+00 -8.77898150E-01
C  +5.59219442E+00 +7.17008892E+00 -2.08729375E+00
H  +5.93198522E+00 +5.80481546E+00 -4.44051777E-01
H  +4.74549834E+00 +7.06923014E+00 -1.06980555E-01
C  +6.49180936E+00 +6.32816983E+00 -3.02660611E+00
C  +4.43002668E+00 +7.82528975E+00 -2.85902715E+00
H  +7.52768010E+00 +6.36217536E+00 -2.64740500E+00
H  +6.50560856E+00 +6.79747892E+00 -4.02149691E+00
H  +4.86982007E+00 +8.52978184E+00 -3.58103418E+00
```

H +3.82723455E+00 +8.41642977E+00 -2.15079694E+00
C +6.45209317E+00 +8.31666784E+00 -1.53417898E+00
H +6.94000563E+00 +8.86976248E+00 -2.34988976E+00
H +7.24106215E+00 +7.93262291E+00 -8.71654379E-01
H +5.84200043E+00 +9.02828396E+00 -9.60629894E-01
H +3.07122447E+00 +5.87985361E+00 -1.13041489E+00
N +4.26151058E+00 +2.51002571E+00 -3.04916525E+00
H +5.18957101E+00 +2.44567237E+00 -3.53014315E+00
H +4.39440062E+00 +2.19450413E+00 -2.08859143E+00
N +6.05008020E+00 +4.93375910E+00 -3.19356798E+00
H +6.49463218E+00 +4.44255633E+00 -3.99656962E+00
H +6.34527903E+00 +4.38911835E+00 -2.37960283E+00

$[Fe(tame)_2]Cl_2$ (LS) PBEPBE-GD3BJMod/cc-pVTZ
Cl +4.05212349E+00 +6.75076216E+00 +1.16923095E+00
Cl +4.45388128E+00 +3.84286001E+00 -6.01184991E+00
Fe +4.25300263E+00 +5.29680868E+00 -2.42130982E+00
N +4.37728521E+00 +4.28655289E+00 -6.72857401E-01
H +3.41417900E+00 +4.03290519E+00 -4.26923785E-01
H +4.63922374E+00 +4.92035059E+00 +1.11214270E-01
N +2.64026792E+00 +6.22634657E+00 -1.58991100E+00
H +2.81203202E+00 +6.14247269E+00 -5.63828779E-01
H +1.75262532E+00 +5.75833303E+00 -1.79418716E+00
N +3.23785724E+00 +3.69912638E+00 -3.14336015E+00
H +3.48996276E+00 +3.67663818E+00 -4.16354750E+00
C +3.60268845E+00 +2.37195493E+00 -2.56954834E+00
C +5.04675284E+00 +2.30460061E+00 -2.02962898E+00
H +3.48225575E+00 +1.59500859E+00 -3.34172949E+00
H +2.90525389E+00 +2.12325799E+00 -1.75382091E+00
C +6.03588646E+00 +2.89762016E+00 -3.03940720E+00
C +5.16735210E+00 +3.04305712E+00 -6.83643489E-01
H +5.90583302E+00 +2.40418437E+00 -4.01464405E+00
H +7.05701984E+00 +2.68775567E+00 -2.68628990E+00
H +6.22144240E+00 +3.29789712E+00 -4.98168463E-01
H +4.84894873E+00 +2.37537254E+00 +1.35686816E-01
C +5.41420573E+00 +8.31000535E-01 -1.81953984E+00
H +6.39732951E+00 +7.36879451E-01 -1.33599433E+00
H +5.45482404E+00 +2.92961113E-01 -2.77684965E+00
H +4.67634681E+00 +3.29316765E-01 -1.17689632E+00
H +2.21570500E+00 +3.78416875E+00 -3.12122735E+00
N +5.26814117E+00 +6.89449717E+00 -1.69925734E+00
H +6.29029435E+00 +6.80946619E+00 -1.72139404E+00
C +4.90329372E+00 +8.22167087E+00 -2.27305713E+00
C +3.45923519E+00 +8.28901207E+00 -2.81299418E+00
H +5.60073372E+00 +8.47039113E+00 -3.08877272E+00
H +5.02370441E+00 +8.99860783E+00 -1.50086309E+00
C +3.33865932E+00 +7.55055715E+00 -4.15898265E+00
C +2.47009170E+00 +7.69598435E+00 -1.80323077E+00
H +3.65707243E+00 +8.21824547E+00 -4.97830621E+00
H +2.28457319E+00 +7.29571250E+00 -4.34447385E+00
H +1.44896278E+00 +7.90581962E+00 -2.15637772E+00
H +2.60010960E+00 +8.18944036E+00 -8.27999447E-01
C +3.09177300E+00 +9.76260957E+00 -3.02308669E+00
H +2.10865336E+00 +9.85672295E+00 -3.50664203E+00
H +3.82963428E+00 +1.02642991E+01 -3.66572305E+00
H +3.05114098E+00 +1.03006487E+01 -2.06577731E+00
H +5.01603788E+00 +6.91697976E+00 -6.79068195E-01
N +5.86573847E+00 +4.36726602E+00 -3.25270284E+00
H +5.69399888E+00 +4.45116066E+00 -4.27878622E+00
H +6.75338325E+00 +4.83526231E+00 -3.04839527E+00
N +4.12873203E+00 +6.30706549E+00 -4.16976041E+00
H +3.86681037E+00 +5.67326814E+00 -4.95383984E+00
H +5.09184075E+00 +6.56072052E+00 -4.41567721E+00

$[Fe(tame)_2]^{+2}$ (HS) OPBE-PCM(water)/cc-pVTZ
Fe +1.03793217E-01 +1.60635435E-02 +9.75354712E-02
N +5.38164715E-02 +1.37079849E-01 +2.31787492E+00
H +7.32165164E-01 +8.55645325E-01 +2.55483538E+00
H +3.96365655E-01 -6.96490940E-01 +2.78628008E+00

N +2.27242825E+00 +4.52937894E-01 -5.36102709E-02
H +2.70657877E+00 +6.32761675E-01 +8.46773780E-01
H +2.35591460E+00 +1.33385839E+00 -5.54486161E-01
N -4.85231002E-01 +2.16327270E+00 +1.95705531E-01
H -1.03991462E+00 +2.33582872E+00 -6.37693763E-01
C -1.27724833E+00 +2.54991082E+00 +1.38354592E+00
C -2.10588200E+00 +1.41412766E+00 +2.01381333E+00
H -1.96021126E+00 +3.36787833E+00 +1.12529130E+00
H -5.89853974E-01 +2.94759021E+00 +2.13702616E+00
C -2.86907527E+00 +6.03135335E-01 +9.51885168E-01
C -1.24535638E+00 +5.08365878E-01 +2.91154191E+00
H -3.27260284E+00 +1.29126301E+00 +2.02315273E-01
H -3.73142388E+00 +1.29531044E-01 +1.43702307E+00
H -1.79710579E+00 -4.12636171E-01 +3.12662156E+00
H -1.09437709E+00 +1.01086558E+00 +3.87536603E+00
C -3.15592526E+00 +2.07990722E+00 +2.90954813E+00
H -3.70991901E+00 +1.33169099E+00 +3.48508673E+00
H -3.87395200E+00 +2.64872242E+00 +2.31003349E+00
H -2.67950624E+00 +2.76888640E+00 +3.61411797E+00
H +2.98534221E-01 +2.80460524E+00 +1.15169208E-01
N +6.92833563E-01 -2.13114395E+00 -6.41956000E-04
H -9.09281545E-02 -2.77248327E+00 +7.98737789E-02
C +1.48487293E+00 -2.51776212E+00 -1.18847391E+00
C +2.31351455E+00 -1.38196702E+00 -1.81871096E+00
H +7.97491583E-01 -2.91543152E+00 -1.94197114E+00
H +2.16783343E+00 -3.33573228E+00 -9.30221193E-01
C +1.45299881E+00 -4.76195990E-01 -2.71644121E+00
C +3.07668167E+00 -5.70983826E-01 -7.56756068E-01
H +1.30206321E+00 -9.78665685E-01 -3.68028779E+00
H +2.00473152E+00 +4.44827278E-01 -2.93147198E+00
H +3.93904202E+00 -9.73728780E-02 -1.24186651E+00
H +3.48019184E+00 -1.25911690E+00 -7.18163200E-03
C +3.36357907E+00 -2.04772853E+00 -2.71443365E+00
H +3.91758058E+00 -1.29950102E+00 -3.28995027E+00
H +2.88717977E+00 -2.73670109E+00 -3.41902354E+00
H +4.08159621E+00 -2.61654830E+00 -2.11491201E+00
H +1.24750423E+00 -2.30370490E+00 +8.32765039E-01
N -2.06484327E+00 -4.20794697E-01 +2.48726301E-01
H -2.49902119E+00 -6.00626132E-01 -6.51643021E-01
H -2.14831712E+00 -1.30170987E+00 +7.49613731E-01
N +1.53797830E-01 -1.04965693E-01 -2.12280211E+00
H -1.88787359E-01 +7.28579725E-01 -2.59122653E+00
H -5.24507112E-01 -8.23572457E-01 -2.35976359E+00

$[Fe(tame)_2]^{+2}$ (LS) OPBE-PCM(water)/cc-pVTZ
Fe +3.07473625E-02 +8.23019290E-03 +3.07387211E-02
N +1.08868551E-02 +2.04150171E-01 +2.03797400E+00
H +7.76538976E-01 +8.21868040E-01 +2.29955683E+00
H +2.22991699E-01 -6.61580536E-01 +2.53131718E+00
N +2.02557383E+00 +2.63299160E-01 -3.31458902E-02
H +2.42847005E+00 +4.89757405E-01 +8.74195541E-01
H +2.22153546E+00 +1.08913738E+00 -5.95870701E-01
N -2.77580592E-01 +1.99408443E+00 -1.11138162E-01
H -8.03939073E-01 +2.17765837E+00 -9.62937750E-01
C -1.01590917E+00 +2.64509131E+00 +9.97677455E-01
C -1.98505843E+00 +1.68672159E+00 +1.69626674E+00
H -1.57626443E+00 +3.50518869E+00 +6.13092357E-01
H -2.92156959E-01 +3.03334509E+00 +1.72072557E+00
C -2.76140477E+00 +8.69889187E-01 +6.58171610E-01
C -1.22261039E+00 +7.59031487E-01 +2.64799476E+00
H -3.07069663E+00 +1.52261003E+00 -1.63766562E-01
H -3.67709613E+00 +4.76708158E-01 +1.11569310E+00
H -1.86683887E+00 -7.19771542E-02 +2.95158755E+00
H -9.65514390E-01 +1.31057180E+00 +3.56024901E+00
C -2.98119701E+00 +2.51550110E+00 +2.50730221E+00
H -3.63113222E+00 +1.87166596E+00 +3.10789957E+00
H -3.61355156E+00 +3.11404504E+00 +1.84446870E+00
H -2.45728899E+00 +3.19793278E+00 +3.18350413E+00
H +5.84503115E-01 +2.52119246E+00 -2.42639722E-01

N +3.39075299E-01 -1.97762393E+00 +1.72616193E-01
H -5.23008121E-01 -2.50473210E+00 +3.04119172E-01
C +1.07740272E+00 -2.62863150E+00 -9.36199707E-01
C +2.04655145E+00 -1.67026233E+00 -1.63479046E+00
H +3.53649765E-01 -3.01688540E+00 -1.65924699E+00
H +1.63775819E+00 -3.48872878E+00 -5.51614700E-01
C +1.28410264E+00 -7.42573074E-01 -2.58651857E+00
C +2.82289903E+00 -8.53429324E-01 -5.96696668E-01
H +1.02700432E+00 -1.29411478E+00 -3.49877132E+00
H +1.92833146E+00 +8.84343217E-02 -2.89011401E+00
H +3.73858960E+00 -4.60248319E-01 -1.05421975E+00
H +3.13219233E+00 -1.50615007E+00 +2.25241029E-01
C +3.04268925E+00 -2.49904253E+00 -2.44582623E+00
H +3.69262425E+00 -1.85520782E+00 -3.04642429E+00
H +2.51878062E+00 -3.18147439E+00 -3.12202750E+00
H +3.67504404E+00 -3.09758632E+00 -1.78299281E+00
H +8.65434753E-01 -2.16119714E+00 +1.02441534E+00
N -1.96407866E+00 -2.46839224E-01 +9.46221956E-02
H -2.36697343E+00 -4.73297976E-01 -8.12719730E-01
H -2.16004075E+00 -1.07267724E+00 +6.57347116E-01
N +5.06069550E-02 -1.87689623E-01 -1.97649651E+00
H -1.61496928E-01 +6.78041514E-01 -2.46983937E+00
H -7.15046494E-01 -8.05406136E-01 -2.23807868E+00

$[Fe(tame)_2]^{+2}$ (HS) BP86-GD3BJMod/cc-pVTZ

Fe +1.03810636E-01 +1.60744565E-02 +9.75489357E-02
N +3.51984542E-02 +1.16780966E-01 +2.33832509E+00
H +7.10132069E-01 +8.50304459E-01 +2.57602128E+00
H +3.81544619E-01 -7.06844368E-01 +2.83983223E+00
N +2.26904409E+00 +4.78883481E-01 -1.02037239E-01
H +2.74876934E+00 +7.63571942E-01 +7.57630774E-01
H +2.32698323E+00 +1.30517165E+00 -7.06094057E-01
N -5.02122817E-01 +2.16629095E+00 +2.02335857E-01
H -1.11762609E+00 +2.29424449E+00 -6.06221154E-01
C -1.26544478E+00 +2.56413583E+00 +1.43394967E+00
C -2.12799814E+00 +1.43230780E+00 +2.03493191E+00
H -1.91459400E+00 +3.42117986E+00 +1.20341271E+00
H -5.41175329E-01 +2.91491560E+00 +2.18266772E+00
C -2.87678605E+00 +6.41773327E-01 +9.39043189E-01
C -1.28866026E+00 +5.00235704E-01 +2.93425371E+00
H -3.20905331E+00 +1.32555154E+00 +1.45304498E-01
H -3.78688621E+00 +2.06479663E-01 +1.37621939E+00
H -1.84936371E+00 -4.23143394E-01 +3.13840684E+00
H -1.13426649E+00 +9.89695809E-01 +3.90694797E+00
C -3.19297474E+00 +2.09664457E+00 +2.92855246E+00
H -3.74498612E+00 +1.34436861E+00 +3.50806788E+00
H -3.91879954E+00 +2.65804723E+00 +2.32473753E+00
H -2.73043653E+00 +2.79658678E+00 +3.63751063E+00
H +2.42875712E-01 +2.85497475E+00 +5.83971701E-02
N +7.09744121E-01 -2.13414201E+00 -7.23797850E-03
H -3.52543861E-02 -2.82282583E+00 +1.36700735E-01
C +1.47306607E+00 -2.53198689E+00 -1.23885180E+00
C +2.33561942E+00 -1.40015885E+00 -1.83983404E+00
H +7.48796611E-01 -2.88276666E+00 -1.98756985E+00
H +2.12221529E+00 -3.38903092E+00 -1.00831485E+00
C +1.49628152E+00 -4.68086758E-01 -2.73915583E+00
C +3.08440733E+00 -6.09624374E-01 -7.43945322E-01
H +1.34188779E+00 -9.57546838E-01 -3.71185011E+00
H +2.05698493E+00 +4.55292370E-01 -2.94330892E+00
H +3.99450748E+00 -1.74330703E-01 -1.18112153E+00
H +3.41667459E+00 -1.29340259E+00 +4.97933661E-02
C +3.40059603E+00 -2.06449561E+00 -2.73345460E+00
H +3.95260739E+00 -1.31221964E+00 -3.31297002E+00
H +2.93805782E+00 -2.76443782E+00 -3.44241277E+00
H +4.12642083E+00 -2.62589826E+00 -2.12963967E+00
H +1.32524742E+00 -2.26209552E+00 +8.01319020E-01
N -2.06142283E+00 -4.46734535E-01 +2.97135111E-01
H -2.54114808E+00 -7.31422993E-01 -5.62532903E-01
H -2.11936197E+00 -1.27302270E+00 +9.01191930E-01

```
N  +1.72422789E-01 -8.46320991E-02 -2.14322721E+00
H  -1.73923446E-01 +7.38993185E-01 -2.64473438E+00
H  -5.02510767E-01 -8.18155657E-01 -2.38092337E+00
```

[Fe(tame)$_2$]$^{+2}$ (LS) BP86-GD3BJMod/cc-pVTZ

```
Fe +3.07468130E-02 +8.22979150E-03 +3.07382856E-02
N  +1.51379339E-02 +2.28336599E-01 +2.05750079E+00
H  +7.56108267E-01 +8.92500412E-01 +2.30980324E+00
H  +2.67404214E-01 -6.21219688E-01 +2.57732024E+00
N  +2.05172708E+00 +2.66212118E-01 -4.51526791E-02
H  +2.47844989E+00 +5.29935000E-01 +8.51471632E-01
H  +2.24628985E+00 +1.07291610E+00 -6.49457587E-01
N  -2.94932998E-01 +2.01440344E+00 -1.25573335E-01
H  -8.67338628E-01 +2.17810011E+00 -9.61863610E-01
C  -1.01497975E+00 +2.66486931E+00 +1.02742283E+00
C  -2.00730120E+00 +1.70610964E+00 +1.70954272E+00
H  -1.54967702E+00 +3.55702826E+00 +6.71643217E-01
H  -2.64412636E-01 +3.01337931E+00 +1.75059832E+00
C  -2.78946256E+00 +9.04979700E-01 +6.53390253E-01
C  -1.26212098E+00 +7.52609119E-01 +2.66106700E+00
H  -3.07280901E+00 +1.55631016E+00 -1.85219517E-01
H  -3.72471392E+00 +5.30220006E-01 +1.09300729E+00
H  -1.90015840E+00 -1.04752777E-01 +2.91717940E+00
H  -1.03808441E+00 +1.27286743E+00 +3.60317152E+00
C  -3.00524418E+00 +2.53752729E+00 +2.53195321E+00
H  -3.64723586E+00 +1.88954506E+00 +3.14385606E+00
H  -3.65426588E+00 +3.13428285E+00 +1.87691221E+00
H  -2.48069317E+00 +3.22735922E+00 +3.20679435E+00
H  +5.52017553E-01 +2.56836987E+00 -3.02696817E-01
N  +3.56426593E-01 -1.99794385E+00 +1.87049904E-01
H  -4.90523971E-01 -2.55191027E+00 +3.64173379E-01
C  +1.07647332E+00 -2.64840973E+00 -9.65946268E-01
C  +2.06879482E+00 -1.68965007E+00 -1.64806611E+00
H  +3.25906207E-01 -2.99691967E+00 -1.68912178E+00
H  +1.61117055E+00 -3.54056871E+00 -6.10166677E-01
C  +1.32361466E+00 -7.36149502E-01 -2.59959038E+00
C  +2.85095616E+00 -8.88520163E-01 -5.91913593E-01
H  +1.09957816E+00 -1.25640775E+00 -3.54169495E+00
H  +1.96165208E+00 +1.21212419E-01 -2.85570268E+00
H  +3.78620757E+00 -5.13760533E-01 -1.03153056E+00
H  +3.13430250E+00 -1.53985062E+00 +2.46696217E-01
C  +3.06673781E+00 -2.52106773E+00 -2.47047658E+00
H  +3.70872952E+00 -1.87308551E+00 -3.08237941E+00
H  +2.54218681E+00 -3.21089966E+00 -3.14531773E+00
H  +3.71575950E+00 -3.11782329E+00 -1.81543556E+00
H  +9.28832218E-01 -2.16164054E+00 +1.02334018E+00
N  -1.99023348E+00 -2.49752527E-01 +1.06629228E-01
H  -2.41695627E+00 -5.13475316E-01 -7.89995118E-01
H  -2.18479626E+00 -1.05645657E+00 +7.10934050E-01
N  +4.63557040E-02 -2.11877053E-01 -1.99602421E+00
H  -2.05910627E-01 +6.37679200E-01 -2.51584369E+00
H  -6.94614586E-01 -8.76040920E-01 -2.24832664E+00
```

[Fe(tame)$_2$]$^{+2}$ (HS) PBEPBE-GD3BJMod/cc-pVTZ

```
Fe +1.03810637E-01 +1.60744559E-02 +9.75489378E-02
N  +4.07958871E-02 +1.16414372E-01 +2.33744077E+00
H  +7.15270478E-01 +8.48544355E-01 +2.57841098E+00
H  +3.85901085E-01 -7.07702177E-01 +2.83798527E+00
N  +2.26805079E+00 +4.83860655E-01 -1.00299844E-01
H  +2.74718212E+00 +7.66879510E-01 +7.59649510E-01
H  +2.32944734E+00 +1.31006749E+00 -7.03179365E-01
N  -5.01108507E-01 +2.16652786E+00 +1.96719750E-01
H  -1.11640820E+00 +2.29794537E+00 -6.10779470E-01
C  -1.25970611E+00 +2.55885181E+00 +1.42795154E+00
C  -2.12049493E+00 +1.42752161E+00 +2.02829257E+00
H  -1.90940987E+00 +3.41745484E+00 +1.20352620E+00
H  -5.34319050E-01 +2.90892678E+00 +2.17625252E+00
C  -2.86925716E+00 +6.37766646E-01 +9.34050583E-01
C  -1.28198820E+00 +4.97013905E-01 +2.92727154E+00
```

H -3.20029581E+00 +1.32145828E+00 +1.39412957E-01
H -3.78243018E+00 +2.08190645E-01 +1.37117134E+00
H -1.84221795E+00 -4.27044176E-01 +3.13085063E+00
H -1.13304651E+00 +9.85467842E-01 +3.90163861E+00
C -3.18409934E+00 +2.09091796E+00 +2.92107519E+00
H -3.73701133E+00 +1.33966700E+00 +3.50108837E+00
H -3.91065152E+00 +2.65294230E+00 +2.31869092E+00
H -2.72286647E+00 +2.79100710E+00 +3.63076037E+00
H +2.44304468E-01 +2.85421939E+00 +5.38843916E-02
N +7.08729806E-01 -2.13437893E+00 -1.62186770E-03
H -3.66831559E-02 -2.82207047E+00 +1.41213507E-01
C +1.46732740E+00 -2.52670287E+00 -1.23285366E+00
C +2.32811621E+00 -1.39537266E+00 -1.83319470E+00
H +7.41940342E-01 -2.87677785E+00 -1.98115464E+00
H +2.11703118E+00 -3.38530589E+00 -1.00842833E+00
C +1.48960946E+00 -4.64864968E-01 -2.73217366E+00
C +3.07687844E+00 -6.05617690E-01 -7.38952723E-01
H +1.34066778E+00 -9.53318893E-01 -3.70654074E+00
H +2.04983919E+00 +4.59193134E-01 -2.93575274E+00
H +3.99005145E+00 -1.76041675E-01 -1.17607349E+00
H +3.40791711E+00 -1.28930932E+00 +5.56848980E-02
C +3.39172062E+00 -2.05876901E+00 -2.72597733E+00
H +3.94463259E+00 -1.30751804E+00 -3.30599051E+00
H +2.93048776E+00 -2.75885815E+00 -3.43566251E+00
H +4.11827281E+00 -2.62079334E+00 -2.12359306E+00
H +1.32402951E+00 -2.26579642E+00 +8.05877345E-01
N -2.06042952E+00 -4.51711710E-01 +2.95397716E-01
H -2.53956085E+00 -7.34730565E-01 -5.64551638E-01
H -2.12182608E+00 -1.27791855E+00 +8.98277239E-01
N +1.66825361E-01 -8.42654861E-02 -2.14234289E+00
H -1.78279884E-01 +7.39851034E-01 -2.64288741E+00
H -5.07649195E-01 -8.16395509E-01 -2.38331307E+00

$[Fe(tame)_2]^{+2}$ (LS) PBEPBE-GD3BJMod/cc-pVTZ
Fe +3.07468094E-02 +8.22978870E-03 +3.07382837E-02
N +1.75992702E-02 +2.27791265E-01 +2.05694670E+00
H +7.58714849E-01 +8.90391848E-01 +2.31161721E+00
H +2.68467424E-01 -6.22087468E-01 +2.57646959E+00
N +2.05079774E+00 +2.68531967E-01 -4.45340690E-02
H +2.47740665E+00 +5.30876361E-01 +8.52289627E-01
H +2.24752640E+00 +1.07555831E+00 -6.47160106E-01
N -2.94105788E-01 +2.01375663E+00 -1.27920916E-01
H -8.65166730E-01 +2.17981935E+00 -9.64269510E-01
C -1.01194286E+00 +2.65995805E+00 +1.02359713E+00
C -2.00250669E+00 +1.70213058E+00 +1.70560575E+00
H -1.54731688E+00 +3.55329376E+00 +6.70836198E-01
H -2.61558287E-01 +3.00963050E+00 +1.74674929E+00
C -2.78397110E+00 +9.01277099E-01 +6.51283426E-01
C -1.25760577E+00 +7.50396193E-01 +2.65627812E+00
H -3.06850757E+00 +1.55253901E+00 -1.87277627E-01
H -3.72062397E+00 +5.29466868E-01 +1.09119920E+00
H -1.89574821E+00 -1.06823178E-01 +2.91358684E+00
H -1.03667773E+00 +1.27087253E+00 +3.59935960E+00
C -2.99923860E+00 +2.53252852E+00 +2.52699877E+00
H -3.64244382E+00 +1.88581172E+00 +3.13899047E+00
H -3.64858594E+00 +3.13038131E+00 +1.87325711E+00
H -2.47610890E+00 +3.22246129E+00 +3.20286219E+00
H +5.52948336E-01 +2.56757444E+00 -3.03704829E-01
N +3.55599378E-01 -1.99729705E+00 +1.89397478E-01
H -4.91454756E-01 -2.55111485E+00 +3.65181393E-01
C +1.07343643E+00 -2.64349846E+00 -9.62120581E-01
C +2.06400031E+00 -1.68567101E+00 -1.64412913E+00
H +3.23051845E-01 -2.99317084E+00 -1.68527278E+00
H +1.60881039E+00 -3.53683422E+00 -6.09359684E-01
C +1.31909946E+00 -7.33936563E-01 -2.59480150E+00
C +2.84546469E+00 -8.84817571E-01 -5.89806748E-01
H +1.09817150E+00 -1.25441283E+00 -3.53788305E+00
H +1.95724191E+00 +1.23282842E-01 -2.85211009E+00
H +3.78211763E+00 -5.13007420E-01 -1.02972243E+00

```
H  +3.13000102E+00 -1.53607948E+00 +2.48754354E-01
C  +3.06073224E+00 -2.51606895E+00 -2.46552213E+00
H  +3.70393749E+00 -1.86935216E+00 -3.07751380E+00
H  +2.53760256E+00 -3.20600171E+00 -3.14138557E+00
H  +3.71007955E+00 -3.11392175E+00 -1.81178045E+00
H  +9.26660325E-01 -2.16335979E+00 +1.02574606E+00
N  -1.98930414E+00 -2.52072373E-01 +1.06010613E-01
H  -2.41591303E+00 -5.14416656E-01 -7.90813122E-01
H  -2.18603281E+00 -1.05909878E+00 +7.08636551E-01
N  +4.38943688E-02 -2.11331723E-01 -1.99547013E+00
H  -2.06973841E-01 +6.38546974E-01 -2.51499306E+00
H  -6.97221158E-01 -8.73932367E-01 -2.25014063E+00
```

References

[1] C.-J. Qin, L. James, J. D. Chartres, L. J. Alcock, K. J. Davis, A. C. Willis, A. M. Sargeson, P. V. Bernhardt , S. F. Ralph, *Inorg. Chem.* **2011**, *50*, 9131-9140.

[2] M. J. Riley, E. R. Krausz , A. Stanco, *J. Inorg. Biochem.* **2003**, *96*, 217.

[3] V. Dyadkin, P. Pattison, V. Dmitriev , D. Chernyshov, *J. Synchrotron Radiat.* **2016**, *23*, 825-829.

[4] G. M. Sheldrick, *Acta Crystallogr. A* **2008**, *64*, 112-122.

[5] L. Farrugia, *J. Appl. Crystallogr.* **1997**, *30*, 565.

[6] A. Hauser, *J. Chem. Phys.* **1991**, *94*, 2741-2748.

[7] C. P. Slichter , H. G. Drickamer, *J. Chem. Phys.* **1972**, *56*, 2142-2160.

[8] H. Bolvin , O. Kahn, *Chem. Phys.* **1995**, *192*, 295-305.

[9] M. J. Frisch, G. W. Trucks, H. B. Schlegel, G. E. Scuseria, M. A. Robb, J. R. Cheeseman, G. Scalmani, V. Barone, B. Mennucci, G. A. Petersson, H. Nakatsuji, M. Caricato, X. Li, H. P. Hratchian, A. F. Izmaylov, J. Bloino, G. Zheng, J. L. Sonnenberg, M. Hada, M. Ehara, K. Toyota, R. Fukuda, J. Hasegawa, M. Ishida, T. Nakajima, Y. Honda, O. Kitao, H. Nakai, T. Vreven, J. A. Montgomery, J. E. Peralta, F. Ogliaro, M. Bearpark, J. J. Heyd, E. Brothers, K. N. Kudin, V. N. Staroverov, T. Keith, R. Kobayashi, J. Normand, K. Raghavachari, A. Rendell, J. C. Burant, S. S. Iyengar, J. Tomasi, M. Cossi, N. Rega, J. M. Millam, M. Klene, J. E. Knox, J. B. Cross, V. Bakken, C. Adamo, J. Jaramillo, R. Gomperts, R. E. Stratmann, O. Yazyev, A. J. Austin, R. Cammi, C. Pomelli, J. W. Ochterski, R. L. Martin, K. Morokuma, V. G. Zakrzewski, G. A. Voth, P. Salvador, J. J. Dannenberg, S. Dapprich, A. D. Daniels, O. Farkas, J. B. Foresman, J. V. Ortiz, J. Cioslowski , D. J. Fox in *Gaussian 09, Revision D.01, Gaussian, Inc., Wallingford CT*, *Vol.* **2013**.

[10] a) A. D. Becke, *J. Chem. Phys.* **1993**, *98*, 5648-5652; b) C. Lee, W. Yang , R. G. Parr, *Phys. Rev. B.* **1988**, *37*, 785-789; c) S. H. Vosko, L. Wilk , M. Nusair, *Can. J. Phys.* **1980**, *58*, 1200-1211; d) P. J. Stephens, F. J. Devlin, C. F. Chabalowski , M. J. Frisch, *J. Phys. Chem.* **1994**, *98*, 11623-11627.

[11] a) N. C. Handy , A. J. Cohen, *Mol. Phys.* **2001**, *99*, 403-412; b) J. P. Perdew, K. Burke , M. Ernzerhof, *Phys. Rev. Lett.* **1996**, *77*, 3865-3868.

[12] J. P. Perdew, K. Burke , M. Ernzerhof, *Phys. Rev. Lett.* **1997**, *78*, 1396-1396.

[13] a) A. D. Becke, *Phys. Rev. A* **1988**, *38*, 3098-3100; b) J. P. Perdew, *Phys. Rev. B.* **1986**, *33*, 8822-8824.

[14] S. Grimme, S. Ehrlich , L. Goerigk, *J. Comput. Chem.* **2011**, *32*, 1456-1465.

[15] D. G. A. Smith, L. A. Burns, K. Patkowski , C. D. Sherrill, *J. Phys. Chem. Lett.* **2016**, *7*, 2197-2203.

[16] a) M. Swart, *J. Chem. Theory Comput.* **2008**, *4*, 2057-2066; b) R. K. Hocking, R. J. Deeth , T. W. Hambley, *Inorg. Chem.* **2007**, *46*, 8238-8244; c) M. Foscato, B. J. Houghton, G. Occhipinti, R. J. Deeth , V. R. Jensen, *J. Chem. Inf. Model.* **2015**, *55*, 1844-1856.

[17] a) J. Tomasi, B. Mennucci , R. Cammi, *Chem. Rev.* **2005**, *105*, 2999-3094; b) F. M. Floris, J. Tomasi , J. L. P. Ahuir, *J. Comput. Chem.* **1991**, *12*, 784-791; c) R. A. Pierotti, *Chem. Rev.* **1976**, *76*, 717-726.

[18] a) T. H. Dunning, *J. Chem. Phys.* **1989**, *90*, 1007-1023; b) D. E. Woon , T. H. Dunning, *J. Chem. Phys.* **1994**, *100*, 2975-2988; c) N. B. Balabanov , K. A. Peterson, *J. Chem. Phys.* **2005**, *123*, 064107.

[19] S. A. Dalrymple , G. K. H. Shimizu, *J. Mol. Struct.* **2006**, *796*, 95-106.

[20] *Nomenclature of Inorganic Chemistry: IUPAC Recommendations 2005*, RSC Publishing, **2005**, p. 193.

[21] K. P. Kepp, *Coord. Chem. Rev.* **2013**, *257*, 196-209.

[22] R. J. Deeth, C. M. Handley , B. J. Houghton in *Spin-Crossover Materials: Properties and Applications*, (Ed. M. A. Halcrow), John Wiley & Sons Ltd, Hoboken, **2013**, pp. 443-454.
